# Sequence analyses of Malaysian Indigenous communities reveal historical admixture between Hoabinhian hunter-gatherers and Neolithic farmers

**DOI:** 10.1038/s41598-022-17884-8

**Published:** 2022-08-12

**Authors:** Farhang Aghakhanian, Boon-Peng Hoh, Chee-Wei Yew, Vijay Kumar Subbiah, Yali Xue, Chris Tyler-Smith, Qasim Ayub, Maude E. Phipps

**Affiliations:** 1 MUM Genomics Facility, Monash University Malaysia, 47500 Bandar Sunway, Selangor Darul Ehsan Malaysia; 2 TropMed and Biology Multidisciplinary Platform, Monash University Malaysia, 47500 Bandar Sunway, Selangor Malaysia; 3 Present Address: Department of Medicine, Institute for Global Health and Infectious Diseases, University of North Carolina at Chapel Hill, Chapel Hill, NC USA; 4 Faculty of Medicine and Health Sciences, UCSI University, Jalan Menara Gading, Taman Connaught, 56000 Cheras, Kuala Lumpur Malaysia; 5 Jeffrey Cheah School of Medicine and Health Sciences, Monash University Malaysia, Jalan Lagoon Selatan, 46150 Bandar Sunway, Selangor Malaysia; 6 Biotechnology Research Institute, University Malaysia Sabah, Jalan UMS, 88400 Kota Kinabalu, Sabah Malaysia; 7 Wellcome Sanger Institute, Wellcome Genome Campus, Hinxton, CB10 1SA UK

**Keywords:** Evolution, Genetics

## Abstract

Southeast Asia comprises 11 countries that span mainland Asia across to numerous islands that stretch from the Andaman Sea to the South China Sea and Indian Ocean. This region harbors an impressive diversity of history, culture, religion and biology. Indigenous people of Malaysia display substantial phenotypic, linguistic, and anthropological diversity. Despite this remarkable diversity which has been documented for centuries, the genetic history and structure of indigenous Malaysians remain under-studied. To have a better understanding about the genetic history of these people, especially Malaysian Negritos, we sequenced whole genomes of 15 individuals belonging to five indigenous groups from Peninsular Malaysia and one from North Borneo to high coverage (30X). Our results demonstrate that indigenous populations of Malaysia are genetically close to East Asian populations. We show that present-day Malaysian Negritos can be modeled as an admixture of ancient Hoabinhian hunter-gatherers and Neolithic farmers. We observe gene flow from South Asian populations into the Malaysian indigenous groups, but not into Dusun of North Borneo. Our study proposes that Malaysian indigenous people originated from at least three distinct ancestral populations related to the Hoabinhian hunter-gatherers, Neolithic farmers and Austronesian speakers.

## Introduction

Southeast Asia (SEA) has rich demographic, linguistic, and genetic diversity. The region is home to around 1249 ethnic groups belonging to five language families^1^. Despite this fascinating diversity, the genetic history of the region remains under-studied and several outstanding gaps regarding the peopling of this region by anatomically modern humans (AMH) still exist. The four most-debated issues concerning the history of AMH in SEA relate to 1—The timing of their arrival in SEA; 2—Origins of hunter-gatherer populations in SEA and their relationship to the Hoabinhian culture; 3—Process of transition from foraging to farming lifestyle, and 4—Development of the cultural groups today recognized as Austroasiatic and Austronesian^2,3^. According to archeological and early mitochondrial (mt) DNA investigations, the presence of AMH in SEA dates back to around 70–50 k years ago (kya)^4–8^. Later, genome-wide and ancient DNA studies postulated that the AMH entered the region following the “Out-of-Africa” human migration, perhaps via the southern coastal route, and subsequently spread into East Asia (EA), Papua New Guinea, and Australia^9–13^. Subsequently, migrations from EA during the late-Pleistocene and Holocene, and population movements within the region, have shaped today’s population structure of SEA^10,13–15^. The geographical location of Malaysia, a country that is physically split between mainland Asia and Borneo with significant population diversity, provides us with an opportunity to study the population history in SEA.

Malaysia is divided into a western part comprising Peninsular Malaysia and an eastern part on the Island of Borneo comprising the States of Sarawak and Sabah. Indigenous populations comprise 13.8% of the about 32 million population of Malaysia^16^. The myriad indigenous communities of East and West express high ethno-linguistic and cultural diversity. The indigenous populations of Peninsular Malaysia are known as Orang Asli (“Original People” in the Malay language). They comprise 0.7% of the Peninsular Malaysia population and are divided into 3 major groups including Negrito, Senoi, and Proto-Malay based on their morphological and ethnolinguistic characteristics^17^. Malaysian Negrito are hunter-gatherers who reside in the rain-forests of northern Peninsular Malaysia and are proposed to be descendants of the first settlers of Malaysia^4,5,9,14,18^. They speak the Northern-Aslian dialect of the Austroasiatic (AA) language family, and their tradition involves egalitarianism and a patrilineal descent system. Senoi inhabit the central parts of Peninsular Malaysia. They speak the central and southern dialects of the Aslian language, and they traditionally practice slash-and-burn farming. Proto-Malay speak the Malay dialect of the Austronesian language family. They mainly live in the southern parts of Peninsular Malaysia. Proto-Malay practice farming and rain-forest harvesting and their traditions involve a marked social hierarchy. Each OA group is further subdivided into 6 subgroups, which makes up 18 OA subgroups. In Sarawak, the indigenous people are collectively known as Orang Ulu (“People of up-river land” in Malay) and comprise 40% of Sarawak’s population. The indigenous populations of Sabah make up 58.6% of Sabah’s population and are divided into 39 tribes. Dusun, Murut, Paitan, and Bajau are the major indigenous groups in Sabah^16^.

Early anthropological studies proposed multiple competing theories about the origin of OAs. The “layer-cake” theory postulated that all three OA groups originated outside of Peninsular Malaysia and entered Malaysia at different times^19^. Another theory by Benjamin (1985) proposed an in situ development and diversification of OAs^20^ following the first wave of human migration into Asia. Bellwood (1993) suggested that the ancestors of today Senois are associated with early Austroasiatic agriculturists who entered Peninsular during mid-Holocene era^21^. Later interactions between these Neolithic farmers and local hunter-gatherers (ancestors of Negritos) resulted in language shift in Negritos as well as intermediate phenotypical features in Senois. He suggested that Proto-Malays originated from Austronesian speaking farmers who migrated to Malaysia during “Austronesian expansion” approximately 5–7 KYA. Early mtDNA studies found both haplogroups unique to Peninsular Malaysia, and those stablished in Indochina in OAs which suggest gene flow from neighboring populations in SEA into OAs^4,5,14,22^. These studies identified two haplogroups of M21 and R21 in Negrito and Senoi with TMRCA around 30–50 KYA. Higher frequency of these two ancient haplogroups in Negritos could indicate that they are the most direct descendants of the earliest settlers of Peninsular Malaysia. Proto-Malay mainly harbor N21 and N22 haplogroups which may be associated with Austronesian expansion via Island Southeast Asia^5,14^. Genotyping studies highlighted genetic affinity between Malaysian Negritos, Andamanese and Filipino Negritos. This may represent an ancient link between these populations^18,23^. Whole genome-sequencing showed that Malaysian Negritos has the deepest divergence time from EA compared with the other two OA groups. This study also traced some level of gene flow from South Asia in OAs^24^.

To advance our knowledge of the genetic structure and history of Malaysia’s indigenous people explore their relationship with the ancient hunter-gatherer and agriculturist communities of Malaysia, we performed high-coverage whole-genome analysis of 15 Orang Asli and Orang Ulu individuals including Negritos (Jehai, and Mendriq), Senoi (MahMeri), Proto-Malay (Seletar, and Jakun), and Dusun, and report the results of our analysis here.

## Results

### Population structure

**Figure 1:**
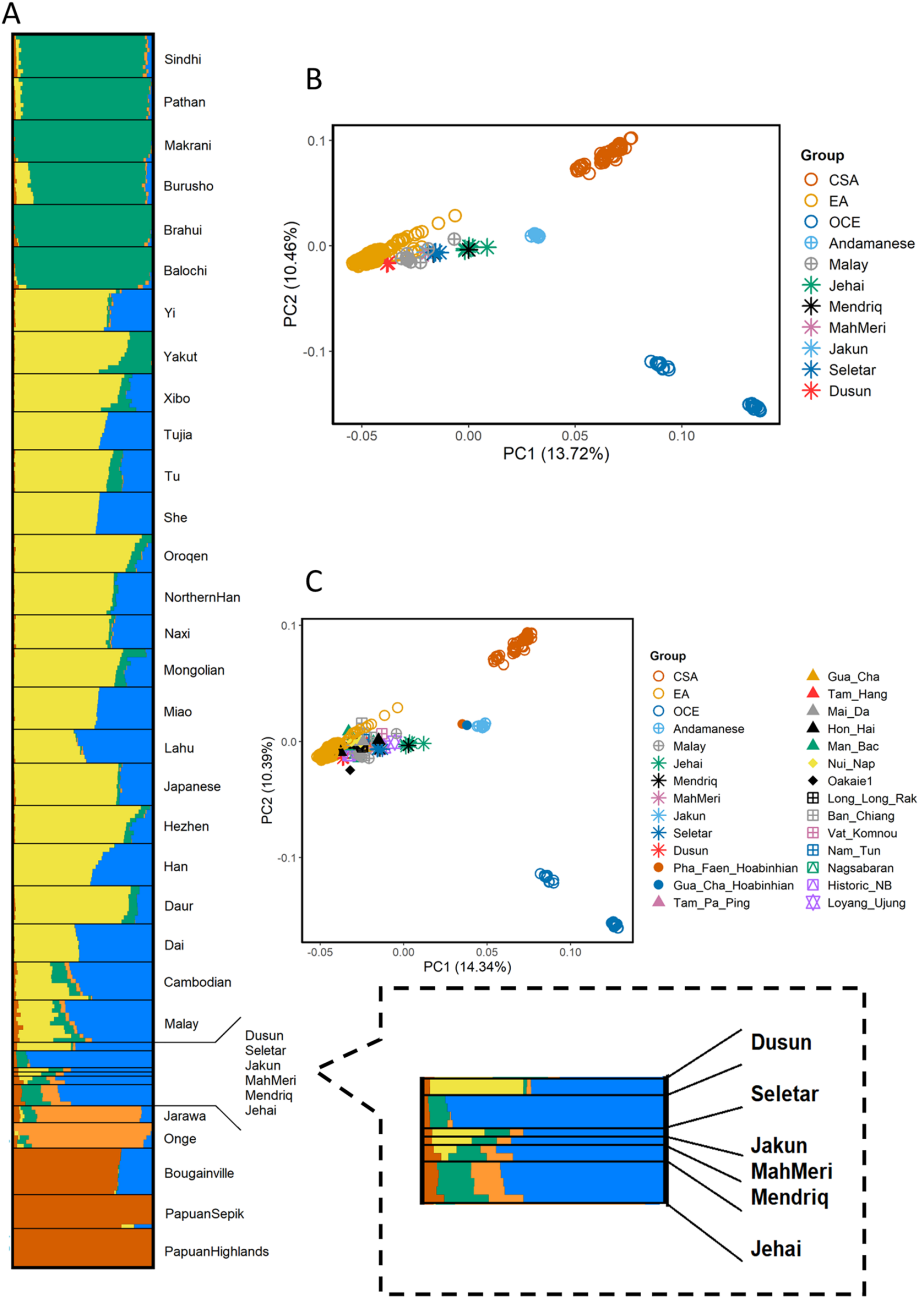
PCA and ADMIXTURE analysis. (**A**) ADMIXTURE analysis results at K = 5 of indigenous Malaysians, Andamanese, Malay, and selected HGDP-CEPH population samplesshowes that the ancestral component related to Southeast Asia (blue) is the most pronounced in OAs while ancestral components related to East Asia (yellow) and South Asia (green) are also present in most of OA groups. (**B**) Global PCA with indigenous Malaysian populations, Andamanese, Malay and selected HGDP-CEPH samples showes that OAs are in general genetically closer to East Asians while Malaysian Negritos have tendency towards Andaman islanders. (**C**) PCA representing ancient Southeast Asian with indigenous Malaysian, Andamanese, Malay and HGDP-CEPH populations from East Asia (EA), Central South Asia (CSA) and Oceania (OCE). Most of OAs positioned between Hoabinhian hunter-gatheres and ancient farmers. Plots are generated using ggplot2 version 3.3.3 package (https://ggplot2.tidyverse.org/) in R version 4.0.4 (https://www.R-project.org/).

To elucidate the genetic history of indigenous people of Malaysia, we sequenced 11 individuals belong to 4 Orang Asli tribes (Fig. S1 and Table S1) at around 30 × coverage using with Illumina HiSeq 2000 platform and included 4 whole genome sequence from Dusun and Mendriq which we published earlier^24^. We used BWA v0.7.12 software to align the sequences to GRCh38 and GATK v3.5.0 for the variant calling. This dataset was merged with Human Genome Diversity Project (HGDP)-CEPH panel data^63^, Andaman Islanders^64^, Malay individuals from Singapore Genome Diversity Project (SSM)^65^. We also constructed a dataset using OAs and ancient AMH samples from southeast Asia (Table S2) to explore the historic link between these groups. We performed Principal Component Analysis (PCA) in order to understand the genetic structure of OAs and their relationship with the surrounding populations. PCA comparing indigenous populations of Malaysia with worldwide populations from the HGDP-CEPH dataset revealed that the indigenous Malaysians are genetically close to East Asian populations (Figs. S2 and S3). This suggests shared ancestors with EA or considerable gene flow between the two groups. On a finer scale, using populations from East, South, and Southeast Asia, OAs especially the Malaysian Negritos, exhibit an affinity towards the South Asians (SA) and Andamanese groups, while Dusun from North Borneo cluster closer to the East Asians (Fig. 1B). This implies a possible admixture between OAs and SA. To explore the relation of Malaysian Negritos with Hoabinhian hunter-gatherers and southeast Asian early farmers we carried out a PCA using ancient SEA samples. The ancient SEA dataset we used in this study includes two Hoabinhian individuals (La368 and Ma911) as well as several Neolithic farmers discovered in archeological sites across Malaysia, Vietnam, Laos and Thailand. PCA with ancient SEAs shows that the ancient samples belonging to the Hoabinhian culture cluster adjacent to the modern-day Andamanese (Figs. 1C and S4). Malaysian Negritos positioned intermediate between the Andamanese/Hoabinhian and EA clusters while the rest of OAs were closer to Neolithic SEA. We conducted ADMIXTURE analysis to infer the genetic ancestry of OAs. In ADMIXTURE analysis of OAs, South, Southeast, and EA populations, the cross-validation score (Fig. S5) proposed that a model with five ancestral components (K = 5) was the best. At K = 5, Seletar (sea nomads) appeared to have a distinct (light blue) ancestral component (presumably the Southeast Asia component), while the Malaysian Negritos displayed a mixture of SEA, Andamanese, and SA components (Figs. 1A and S6). Dusun have the highest EA (yellow) component, which is consistent with the PCA analysis.

### Y-chromosome and mitochondrial DNA haplogroup analysis

We determined the Y-chromosome and mitochondrial DNA haplogroups in the OAs and North Bornean samples (Table S3). For mtDNA, we observed five haplogroups including R21, M21a, M13b1, M17a, and F1a1a in Malaysian Negritos. MahMeri harbored the N22a haplogroup. Jakun carried the E1a2 haplogroup, while all Seletar carried N9a6b. We found two different haplogroups, M7c1c3 and R9c1a, in the Dusuns. The TMRCAs of the R21, M21a, M17a, and F1a1a haplogroups have been dated to 8, 23, 19 and 8 kya, respectively^25^, and have previously been reported In Malaysian^5,14^ and Thai^26^ hunter-gatherers. Haplogroup M13b has been dated to around 31 kya and observed in low frequency in Asia, specifically in Malaysia^4,5^, Tibet^27,28^, and Nepal^29^. The N9a haplogroup is widespread in EA, SA, and SEA^5,30,31^. However, its sub-clade N9a6 appears limited to mainland Southeast Asia (MSEA) and reaches the highest frequency in Peninsular Malaysia^32^. Haplogroup N22a which was observed in MahMeri appears to be restricted to Peninsular Malaysia, although N22 has been recorded in low frequency elsewhere in SEA such as Philippine^33^ and Sumatra^34^. The E1a, M711, R9c1 haplogroups are prevalent in island Southeast Asia (ISEA) and are widely believed to be associated with the Austronesian expansion^35^.

For the Y chromosome, OA harbor the R1a1a1b2a, R2a, K2b, K2b1, and O2b1 haplogroups. The K2b haplogroup and its subclade K2b1, which were observed in Malaysian Negrito and Seletar, have been reported in other SEA Negritos and Oceania^36^. Interestingly, we found haplotypes R2a and R1a1ab in Malaysian Negrito. Haplogroup R2a is mainly present in SA^37,38^ and at lower frequencies in Central, Southwest, and EA^39,40^, while the R1a1a1b, and its sub-clades, comprise the major R1a sub-clades in Central and South Asia^41–43^.

### Estimation of effective population sizes and divergence times

**Figure 2:**
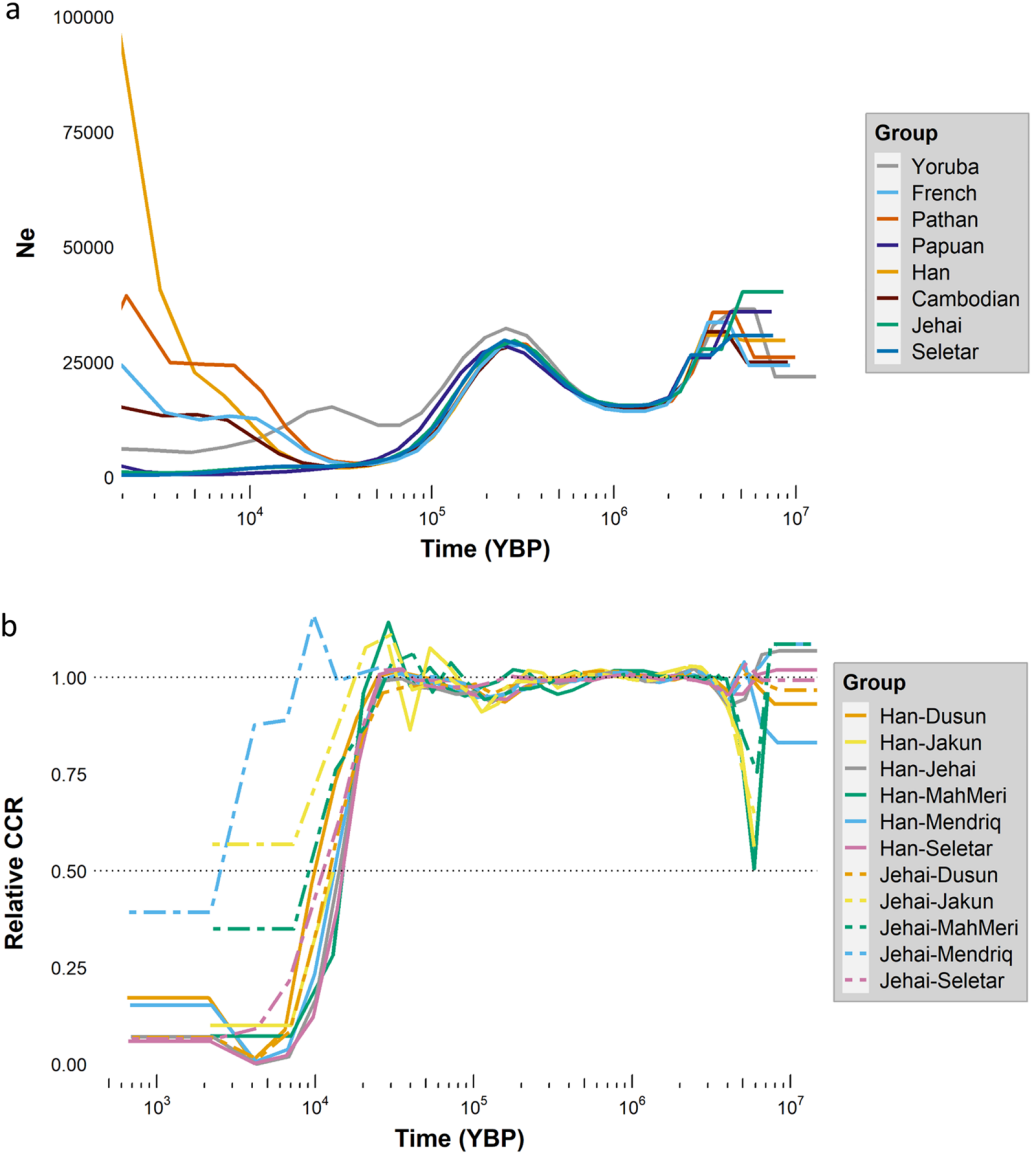
Inference of effective population size (Ne) and population divergence using MSMC2. (**a**) Inference of Ne in Jehai, Seletar and selected HGDP populations using eight haplotypes per population. Pattern of Ne through time in Jehai and Seletar is consistent with Out-of-Africa human migration, however both tribes retained a low Ne after the bottleneck 70–50 KYBP. (**b**) Estimation of divergence time between indigenous Malaysian and Han using four randomly selected haplotypes per population (in case of Mah Meri and Jakun limited to two haplotypes). A relative cross coalescence rate (CCR) around 0.5 heurostically can be used as a split time between the two populations. Plots are generated using ggplot2 version 3.3.3 package (https://ggplot2.tidyverse.org/) in R version 4.0.4 (https://www.R-project.org/).

To estimate the effective population size (Ne) and divergence time in OAs, we carried out a Multiple Sequentially Markovian Coalescent (MSMC2) analysis. In order to have a better resolution, we first included four randomly selected individuals (8 haplotypes) from each population in the MSMC2 analysis. This limited us to only Jehai and Seletar tribes which have sufficient sample size (Figs. 2a and S10). In later stage we tried to include all the tribes in the analysis by recruiting two randomly selected individuals (4 haplotypes) from each population or only 1 individual (2 haplotypes) for Mah Meri and Jakun tribe (Figs. 2b and S9). In general, OAs retained a lower Ne after around 30 kya than neighboring populations. These results could be further supported by the runs of homozygosity (ROH) analysis which revealed long stretches of ROH in OAs (Fig. S11). We found an increase in Ne in Dusun around 6 kya, which possibly coincides with the Austronesian expansion.

For the divergence time, we found that the split between Malaysian Negritos and EA took place around 14–13 kya (Fig. 2b) which is consistent with the results of our previous study using genotyping data^18^. Seletar and Dusun diverged from Han around 10 kya, which is in good agreement with the initial divergence of Austronesian from EA^44^. Overall, the divergences between different Malaysian groups were relatively recent. The divergence between Malaysian Negritos and Austronesians occurred around 12 kya, followed by a split from MahMeri around 9 kya. Jehai and Mendriq (two Negrito tribes) separated from each other approximately 2.6 kya.

### Gene flow between indigenous Malaysian and neighboring populations

**Figure 3:**
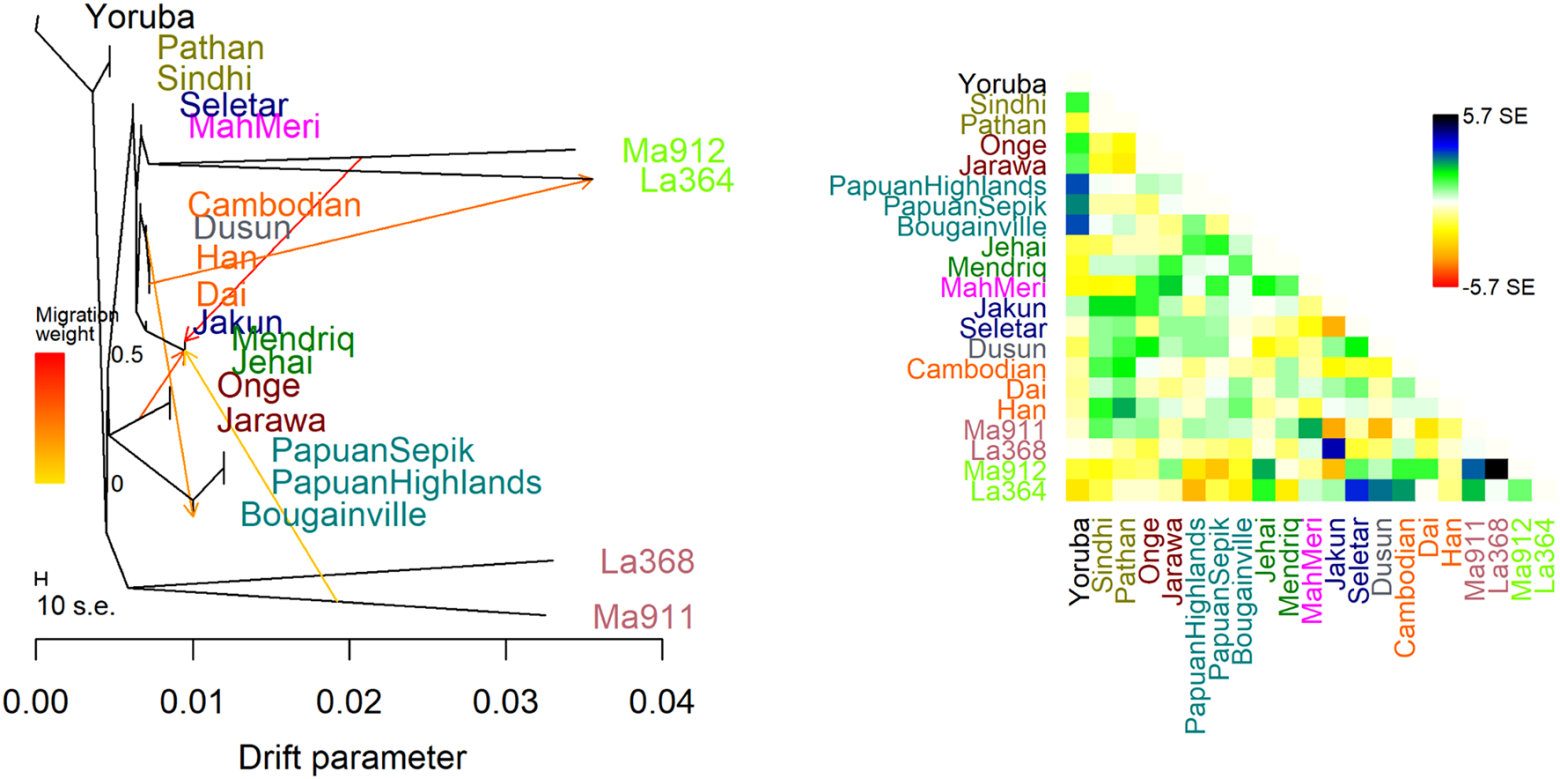
TreeMix maximum likelihood tree with five migration events of indigenous Malaysian and Hobinhian culture and Neolithic farmer ancient Southeast Asia samples. Malaysian Negritos and Jakun make a separate clade while the rest of OAs clustered with East Asians. Results show gene flow events from Malysian Hoabinhian hunther-gatheres (Ma911) and Malaysian Neolithic farmers (Ma912) into modern-day Malaysian Negritos.

To investigate potential gene flow in the history of indigenous Malaysian and modern and ancient populations, we performed a TreeMix analysis (Figs. 3 and S12). For modern populations, TreeMix suggested five migration events. The tree topology revealed that Malaysian Negritos formed a separate cluster while the other Malaysian indigenous groups clustered with EA populations. We identified gene flow between Andamanese and Malaysian Negritos. Our analysis also demonstrated gene flow between Dusun and Melanesian Bougainville. This may reflect the admixture between a population genetically close to today's Dusun in Borneo and a population with Papuan ancestry, attributed to the Austronesian expansion, which has been described by previous studies^45–47^. We also detected gene flow within OA groups, notably from Jehai (Negrito) to Jakun (Proto-Malay), and from MahMeri (Senoi) to Mendriq (Negrito).

TreeMix analysis of Malaysian indigenous and ancient SEA samples revealed similar topology. The Hoabinhian samples clustered separately and next to the Andamanese-Papuan clade, whereas Neolithic SEA clustered with modern EA. Interestingly, our analysis revealed gene flow from two ancient samples from Malaysia, namely Ma911 (Hoabinhian hunter-gatherer) and Ma912 (Neolithic farmer), to the Malaysian Negritos.

**Figure 4:**
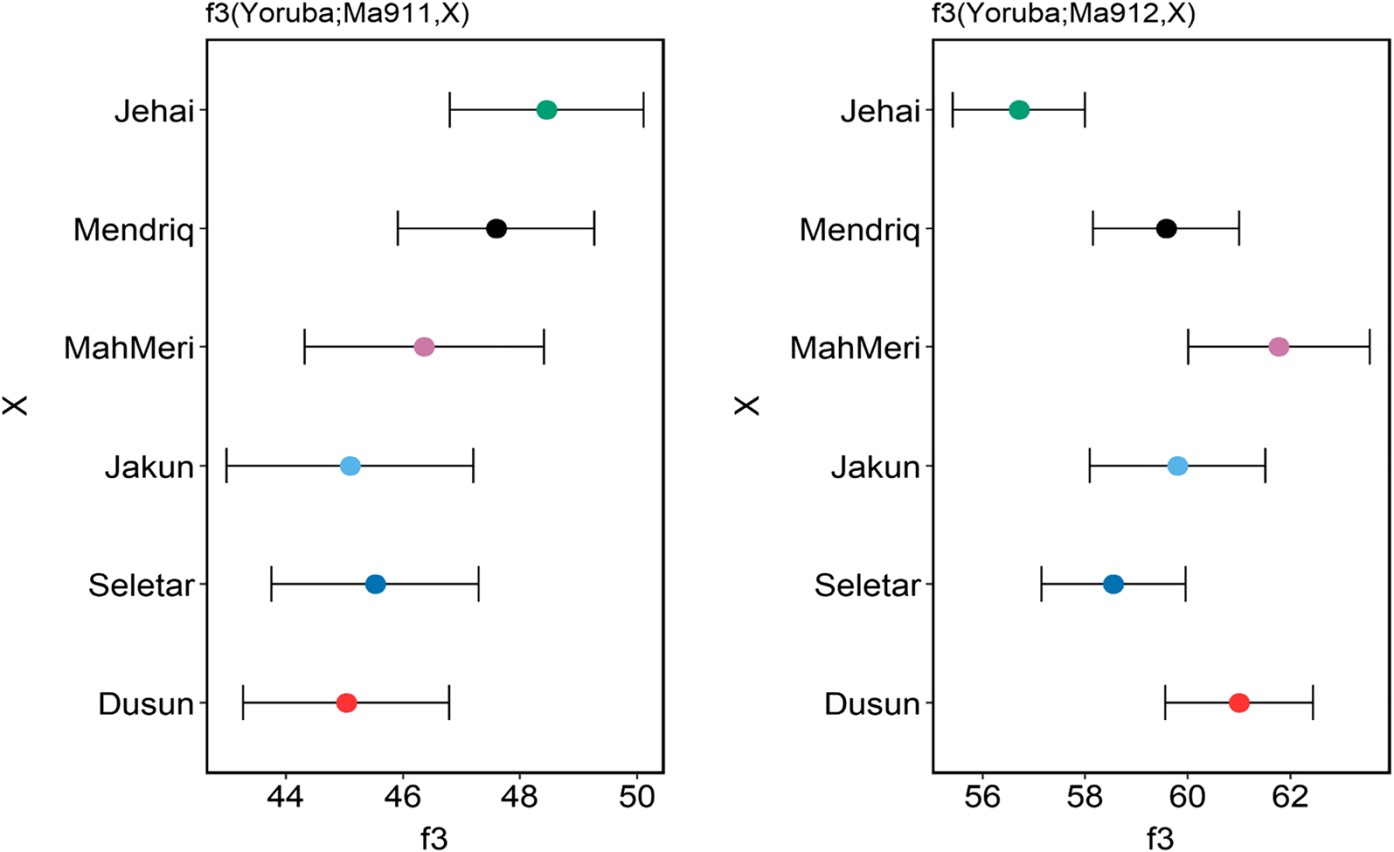
Allele sharing estimation using Outgroup-f3 statistics between Orang Asli and Gua Cha samples. The results indicate that Ma911 (Hoabinhian hunther-gatherer) share more drift with Malaysian Negritos (Jehai and Mendriq), while Ma912 (Neolithic farmer) share more drift with Senois (MahMeri). Plot is generated using ggplot2 version 3.3.3 package (https://ggplot2.tidyverse.org/) in R version 4.0.4 (https://www.R-project.org/).

To better explore the existence of gene flow between OAs and the neighboring populations, within different OA groups and to further confirm the link between modern-day Negritos, Hoabinhian hunter-gatherer and Neolithic farmers, we conducted f4 tests (Table S4). To ascertain links between Malaysian Negritos and Andamanese, we calculated f4(Mbuti, Onge/Jarawa; X, Han), where X denotes the test population. We detected a significant f4-score when setting Jehai as X (Z = − 3.669 and − 3.921 for Onge and Jarawa, respectively); however, f4-scores for Mendriq were not significant. Computing f4(Mbuti, Oceanians; X, Han) displayed a significant f4-score between Dusun and Bougainville (Z = − 3.23). Testing the f4 between different OA groups, we found gene flow between the Malaysian Negritos and their neighboring Jakun (Proto-Malay) and MahMeri (Senoi) groups. However, there was no evidence of gene flow between Malaysian Negritos and Dusun of North Borneo. f4 estimates for ancient Malaysian samples (Ma911 and Ma912) and different OA groups demonstrated significant f4-score only between Ma911 and Malaysian Negrito, while Ma912 had significant f4 with both Malaysian Negritos and Senoi. We computed outgroup-f3 to measure the amount of shared drift between ancient Malaysians and OAs. Interestingly, we noticed that the Hoabinhian Ma911 share the most drift with Malaysian Negritos, while Neolithic farmer Ma912 shared the most drift with Senoi MahMeri (Fig. 4).

## Discussion

Malaysia which lies at the crossroads of South East Asia, has experienced multiple massive human movements over millennia. Archeological and genetic evidence shows that the presence of AMH in Malaysia dates back to at least 40 kya^4–6^. Between 13 to 3 kya Hoabinhian hunter-gatherers occupied the Peninsular. The Hoabinhian culture with a stone tool industry characterized by unifacial pebble tools, are believed to originate from south China and spread throughout mainland Southeast Asia (MSEA) and island Southeast Asia (ISEA)^48^. Since 4 kya, this South East Asian nation also witnessed at least two waves of migration from Neolithic farmers and Austronesian speakers^10,13,49^. These different human migrations and settlements have resulted in Malaysia's rich present-day linguistic and anthropological diversity. Our study leverages new whole-genome sequencing data to dive deeper into the understanding of the genetic structure and history of the indigenous people of this nation.

While indigenous Malaysians are genetically closer to EA populations, consistent with previous studies^18,24^, our new ADMIXTURE analysis revealed traces of South Asian ancestral component in OAs of Peninsular Malaysia. We could not detect this component in Dusun in Borneo. The presence of SA ancestral component in OAs has been previously reported^50,51^. This ancestral component might be attributed to the first wave of human migration into SEA via the southern coastal route or later gene flow from SA during the expansion of Indian culture into Peninsular Malaysia in the first century A.D.^52^. Archeological sites in the state of Perak provide evidence of Hindu civilization. Being on the maritime route between China and South India, the Malay peninsula was involved in this trade. The Bujang Valley, being strategically located at the northwest entrance of the Strait of Malacca as well as facing the Bay of Bengal, was continuously frequented by Chinese and south Indian traders. Such was proven by the discovery of trade ceramics, sculptures, inscriptions and monuments dated from the 5th to fourteenth century CE^53^. More studies are needed to address the source of SA ancestry in Peninsular Malaysia and its absence in Borneo.

Analysis of OAs with ancient DNA from the Gua Cha revealed the contribution of populations genetically close to these samples into the Malaysian Negritos gene pool. The Gua Cha site is a rock shelter in northern Peninsular Malaysia. Based on Sieveking (1954), two archeological phases are recognizable at this site^54^. The Hoabinhian phase when the shelter was used for habitation and occasionally for burial, and the Neolithic phase when it functioned as a cemetery^55^. Radiocarbon dating showed that the Hoabinhian occupied the Gua Cha from 9 kya and later the Neolithic farmers used this site from 3 kya^56^. Our outgroup-f3 analysis is consistent with the archeological findings regarding the transition from hunting-gathering to farming lifestyle in the Gua Cha cave. While the Ma911 (Hoabinhian layer) shared most alleles with the Malaysian Negritos, the Ma912 (Neolithic farmer) was closer to the Senoi agriculturists. Our results confirm that modern Malaysian Negritos have been derived genetically from two ancient populations: the Hoabinhian hunter-gatherers and the Neolithic farmers who originated from South China or MSEA.

Our analysis detected gene flow between different OA tribes, notably between Malaysian Negritos, with MahMeri and Jakun tribes. The admixture between neighboring OA tribes or between OAs and the Malay population has been reported previously^18,57^. For example, Jinam et al. (2013) reported recent admixture between Jehai and their neighboring Malay, whereas such admixture was absent in Kensiu (another Negrito group). We did not find any traces of Negrito or Hoabinhian ancestry in Dusun. Likewise, Yew et al. (2018) reported the absence of Negrito ancestry in North Borneo, Dayak, and Bidayuh populations. Considering the demographical and archeological evidence which supports the presence of Austro-Melanesian people on Borneo Island^58^, the best explanation for the absence of Negrito ancestry in Borneo could be the replacement of initial Austrolo-Melanesian inhabitants of the island by the Austronesians.

Interestingly, all the Seletar samples carried mtDNA N9a6b haplogroup. N96a haplogroup seems to be confined to the ISEA and reaches the highest frequency in Malaysia^32^. Our results are consistent with Jinam et al. (2012) who reported only 4 mtDNA haplogroups (with N9a6b making up of 71% of mtDNA haplogroup frequency) in Seletar. Seletar are sea nomads who live along the strait of Johor (a waterway that separates Malaysia from Singapore). The history of Seletar is not well-documented. They are usually associated with the Orang Laut (“Sea people” in Malay), a conglomerate of sea nomad tribes who occupied the strait of Melaka^59^. Our TreeMix and ROH results indicate that the Seletar are genetically closer to the Austronesian speakers, but they experienced severe genetic drift.

In summary, our study suggests that at least 3 ancestral components were involved in shaping today's indigenous Malaysian populations, the Hoabinhian hunter-gatherers, Neolithic farmers, and Austronesian speakers. We also showed the genetic interaction between different Orang Asli tribes of Peninsular Malaysia.

## Methods

### Samples

This study was reviewed and approved by the Monash University Human Research Ethics Committee (MUHREC), the Department of Orang Asli Development (Jabatan Kemajuan Orang Asli Malaysia, JAKOA), the Research and Ethics Committee of the University of Technology MARA [Ref no: 600-RMI (5/1/6)], and the University of Malaysia Sabah Medical Research Ethics Committee [code: JKEtika 4/10(3)]. All methods were carried out in accordance to the principles of the Declaration of Helsinki. Before sample collection, we paid a courtesy visit to each tribe and obtained approval from the tribe’s chieftain and district offices. We also received approval from the chairperson of the Committee for Village Development and Security for the North Bornean samples.

For this study, we only recruited unrelated volunteer participants above 18 years old who provided written consent. We recruited 11 individuals including 5 Negrito (5 Jehai), 1 Senoi (MahMeri) and 5 Proto-Malay (4 Seletar and 1 Jakun). We collected peripheral blood (6 ml) from each participant and recorded their self-reported ethnicity and family pedigree (Fig. S1).

### DNA extraction, sequencing and variant calling

We extracted the genomic DNA using a modified salting-out method^60^ and the DNeasy Blood and Tissue kit (Qiagen) for the North Bornean samples. We performed sequencing with Illumina HiSeq 2000 at approximately 30 × sequencing coverage and paired-end read length of 100 bp. We also included 2 Negrito Mendriq and 2 North Bornean (Dusun) fastq files from our previous study^24^. We mapped paired-end reads to GRCh38 using the Burrows-Wheeler Aligner^61^ (bwa mem) version 0.7.12. We removed PCR duplicate reads using the Picard MarkDuplicates tool version 1.93 (http://broadinstitute.github.io/picard/). We also performed post-alignment processing, for example, base quality recalibration or local indel realignment. To identify single nucleotide variants (SNVs) and small indels, we used GATK HaplotypeCaller^62^ version 3.5.0 on each individual separately.

### Population genomics

To compare the indigenous Malaysian populations with worldwide populations, we downloaded the Human Genome Diversity Project (HGDP)-CEPH panel data^63^, Andaman Islanders^64^, and Malay individuals from Singapore Genome Diversity Project (SSM)^65^. To investigate the historic relationships between indigenous populations of Malaysia with other populations in Southeast Asia, we downloaded ancient SEA data^13,49^. We used the UCSC LiftOver tool to convert the genome coordinates of Andamanese and Malay and ancient SEA data from hg19 to GRCh38. We constructed two datasets. The first dataset comprised 15 indigenous Malaysians along with 1035 unrelated individuals from HGDP, SSM, and Andamanese dataset. After quality control for each population including missing rate per SNP < 0.05, minor allele frequency > 0.05, and Hardy–Weinberg exact (HWE) test (*P* < 10^−6^), 3,374,375 shared SNVs remained. The second datasets included dataset 1 and 43 ancient SEA samples with 3,347,752 overlapping SNVs.

We performed principal component analysis (PCA) and ancestry estimation to assess the genetic structure of different indigenous groups within Malaysia and also the relationship between these groups and other neighboring populations. For this analysis, we filtered out SNVs with linkage disequilibrium (r^2^ > 0.8) to eliminate the effects of excessive LD. After LD pruning, 812,971 and 806,229 SNVs remained from dataset 1 and dataset 2, respectively. In addition, we normalized the sample size by randomly selecting 10 individuals from each population in the HGDP and SSM datasets (Fig. S4). We used ADMIXTURE^66^ analysis for estimating the ancestry and smartPCA from the EIGENSOFT package^67^ for PCA analysis. For the PCA analysis of dataset2, we first computed the eigenvectors using modern samples and later projected ancient samples onto the first two PCs with “lsqproject” and “shrinkmode” parameters to account for excessive missing data in the ancient samples. We visualized ADMIXTURE results with pong software^68^ (Figs. S5–S8).

We conducted a Multiple Sequentially Markovian Coalescent (MSMC2) analysis^69^ to estimate the effective population size (Ne) changes and divergence time of populations. We generated the VCF and masking files for 4 individuals per population, where applicable, according to the MSMC2 recommended parameters. For phasing the data, we used Eagle version 2.4.1^70^. We also assumed a mutation rate of 1.25 × 10^−8^ per base per human generation and a generation time of 29 years^71^.

To further investigate the relationship between the populations and potential migration events, we inferred a maximum likelihood drift tree using TreeMix version 1.13^15^. We used blocks of 500 SNVs (-k 500) to account for LD and added 5 migration edges sequentially with 100 replications for each migration edge and Yoruba as root population. We examined gene flow between indigenous Malaysians and surrounding populations using ADMIXTOOLS package^72^.

We analyzed the full Y-chromosome and mitochondrial sequences by annotating them with the mutations commonly used for nomenclature. We used HaploGrep^73^ and MitoSuite^74^ to assign the mitochondrial haplogroups. For the Y-chromosome, we called the genotypes with SAMtools/BCFtools version 1.9^75^. We restricted calling to the 10.3 Mb region previously identified to be accessible for short-read sequencing^76^. We used yhaplo^77^ to assign the Y-chromosome haplogroups. We used ggplot2 version 3.3.3 package (https://ggplot2.tidyverse.org) in R version 4.0.4 (https://www.R-project.org/) for visualization^78,79^.

### Ethics approval

This study was approved by the Ministry of Health, Malaysia, Monash University Human Research Ethics Committee (MUHREC), the Department of Orang Asli Development (Jabatan Kemajuan Orang Asli Malaysia, JAKOA), the Research and Ethics Committee of the University of Technology MARA [Ref no: 600-RMI (5/1/6)], and the University of Malaysia Sabah Medical Research Ethics Committee [code: JKEtika 4/10(3)].

### Consent to participate

Both verbal and written informed consent was obtained from all individual participants included in the study.

## Supplementary Information

Supplementary Information.

## Data Availability

The data that support the findings of this study are available through the European Variation Archive with accession number PRJEB48356.
