# Supplementary material for: Sequence analyses of Malaysian Indigenous communities reveal historical admixture between Hoabinhian hunter-gatherers and Neolithic farmers
Source: Sci Rep. 2022 Aug 12;12:13743. doi: 10.1038/s41598-022-17884-8 (PMC9374673; doi:10.1038/s41598-022-17884-8)
Supplement: Supplementary file 1 — Supplementary Information. [file 41598_2022_17884_MOESM1_ESM.docx]

Fig S1: The geographical locations of Indigenous Malaysian groups analyzed in this study. Plot is generated using ggplot2 version 3.3.3 package (https://github.com/tidyverse/ggplot2/releases/ tag/v3.3.3) in R version 4.0.4 (https://www.R-project.org/).


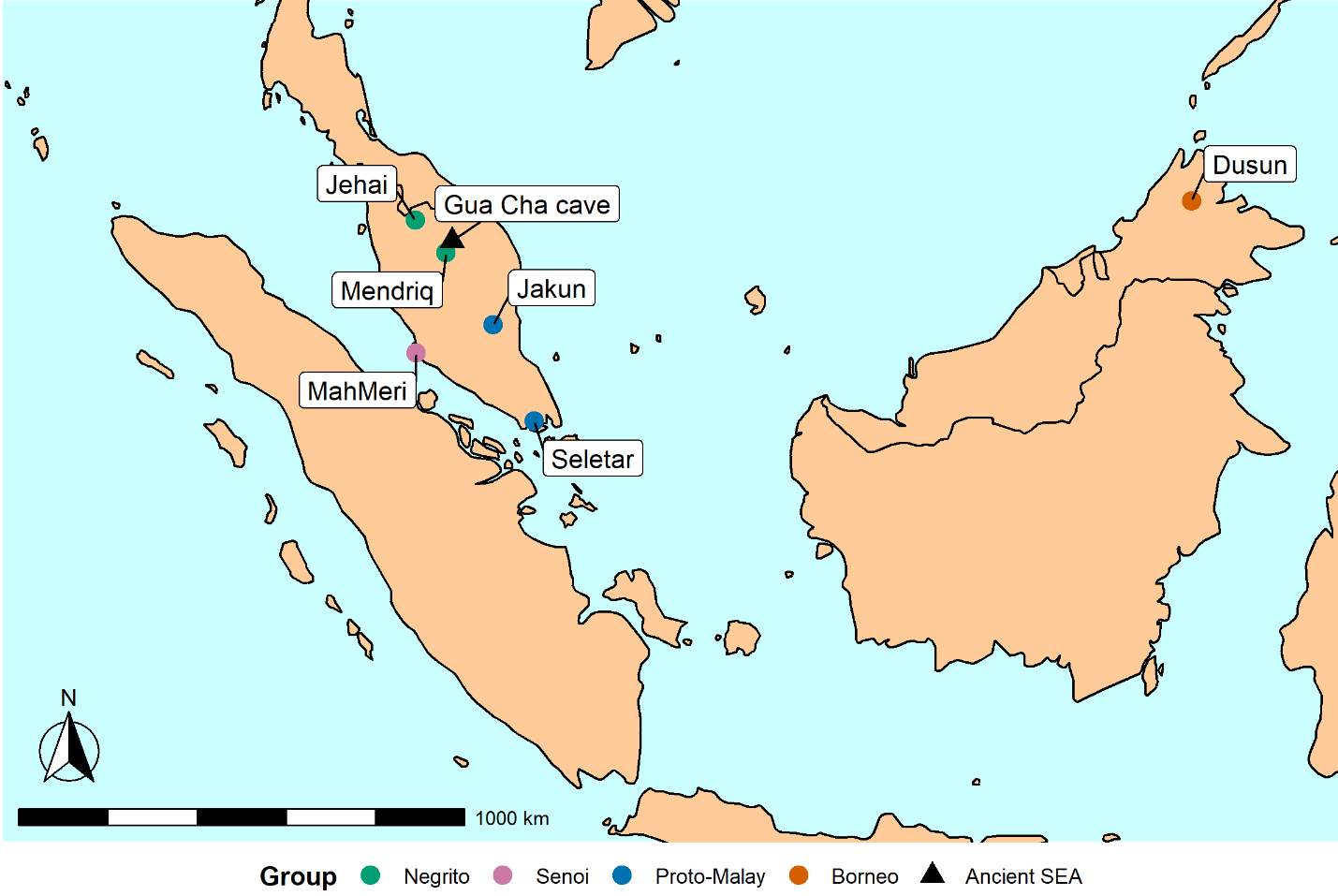


Fig S2: Principal Component Analysis (PCA) of indigenous Malaysian and the HGDP-CEPH global population dataset. (AF: African; AM: American; CSA: Central-South Asian; EA: East Asian; EU: European; ME: Middle Eastern; OCE: Oceanian)


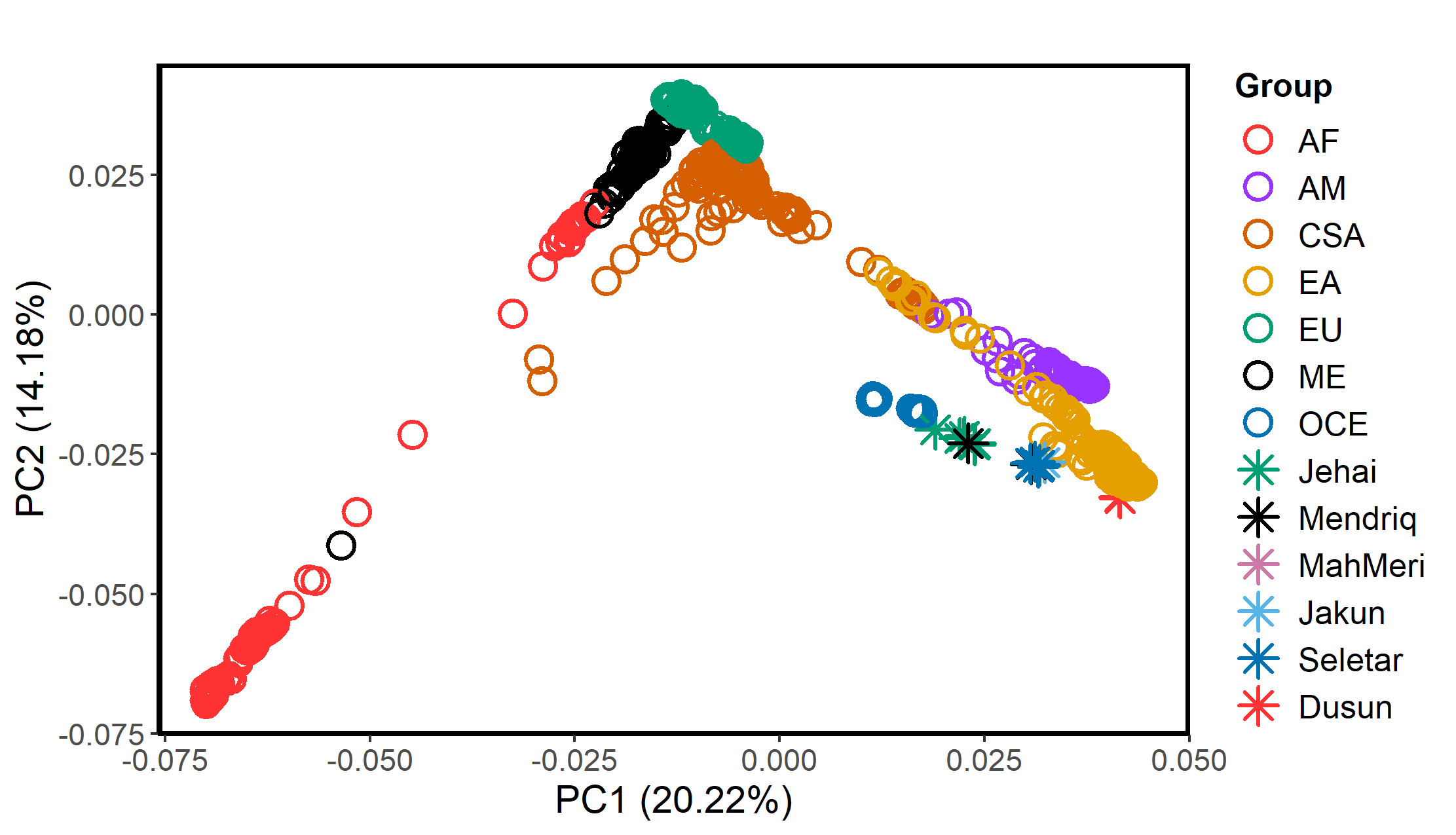


Fig S3: Principal Component Analysis (PCA) of indigenous Malaysian, Andamanese, Malay and EA, CSA, and OCE from HGDP using: **A)** complete dataset, **B)** normalized dataset.

A)


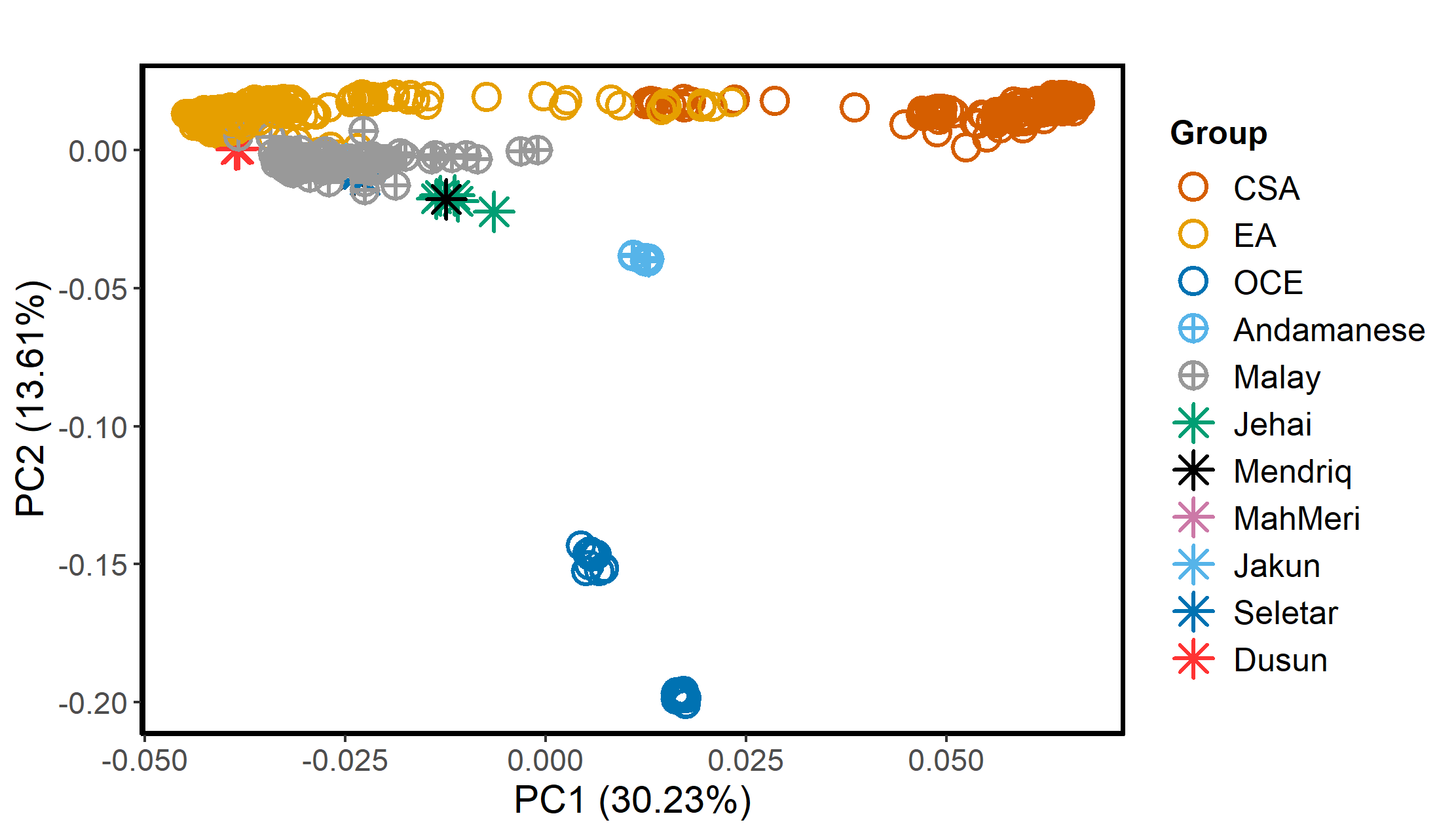


B)


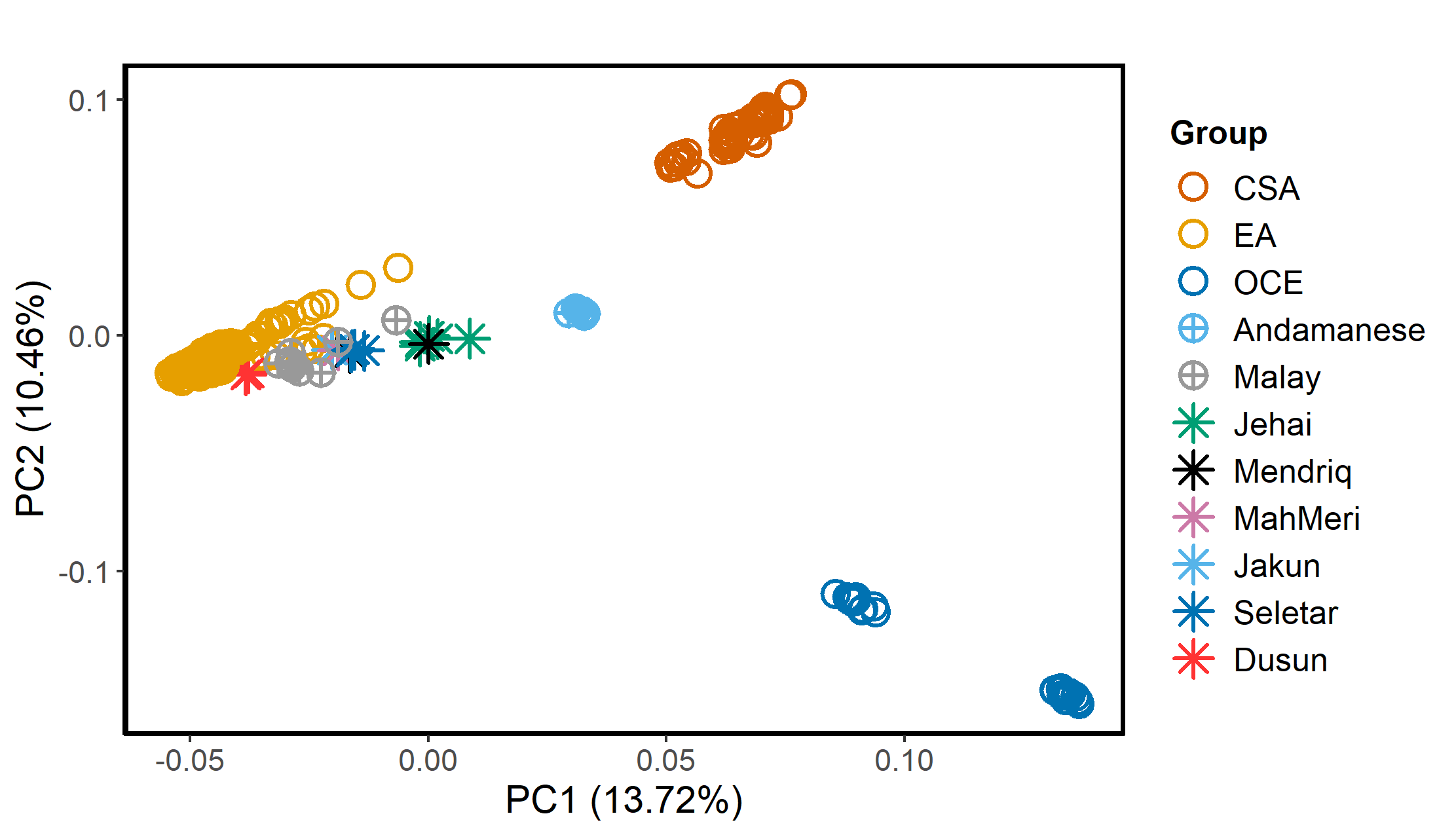


Fig S4: Principal Component Analysis (PCA) of ancient Southeast Asian, indigenous Malaysian, Andamanese, Malay and EA, CSA, and OCE from HGDP using: **A)** complete dataset, **B)** normalized dataset.

A)


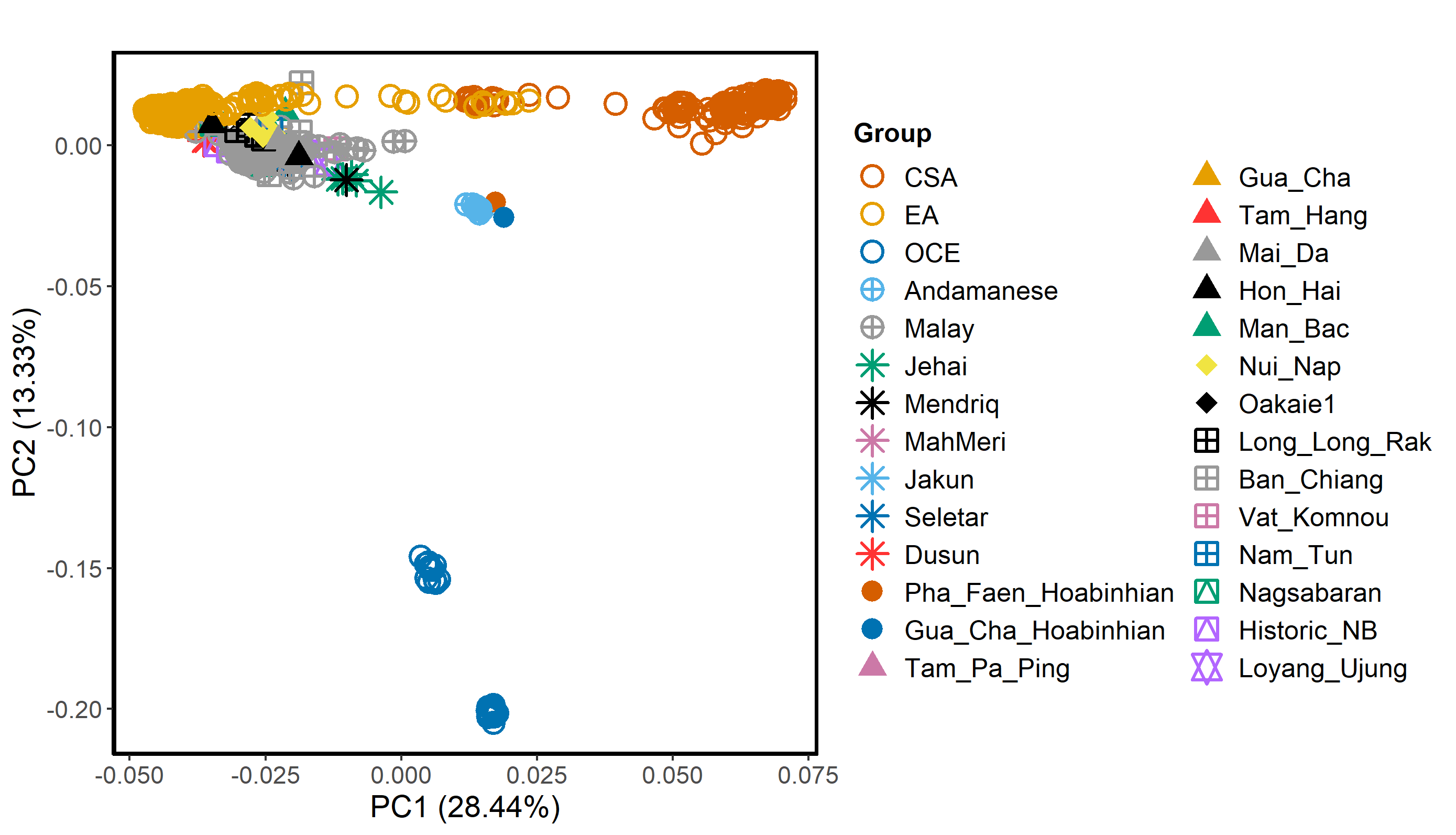


B)


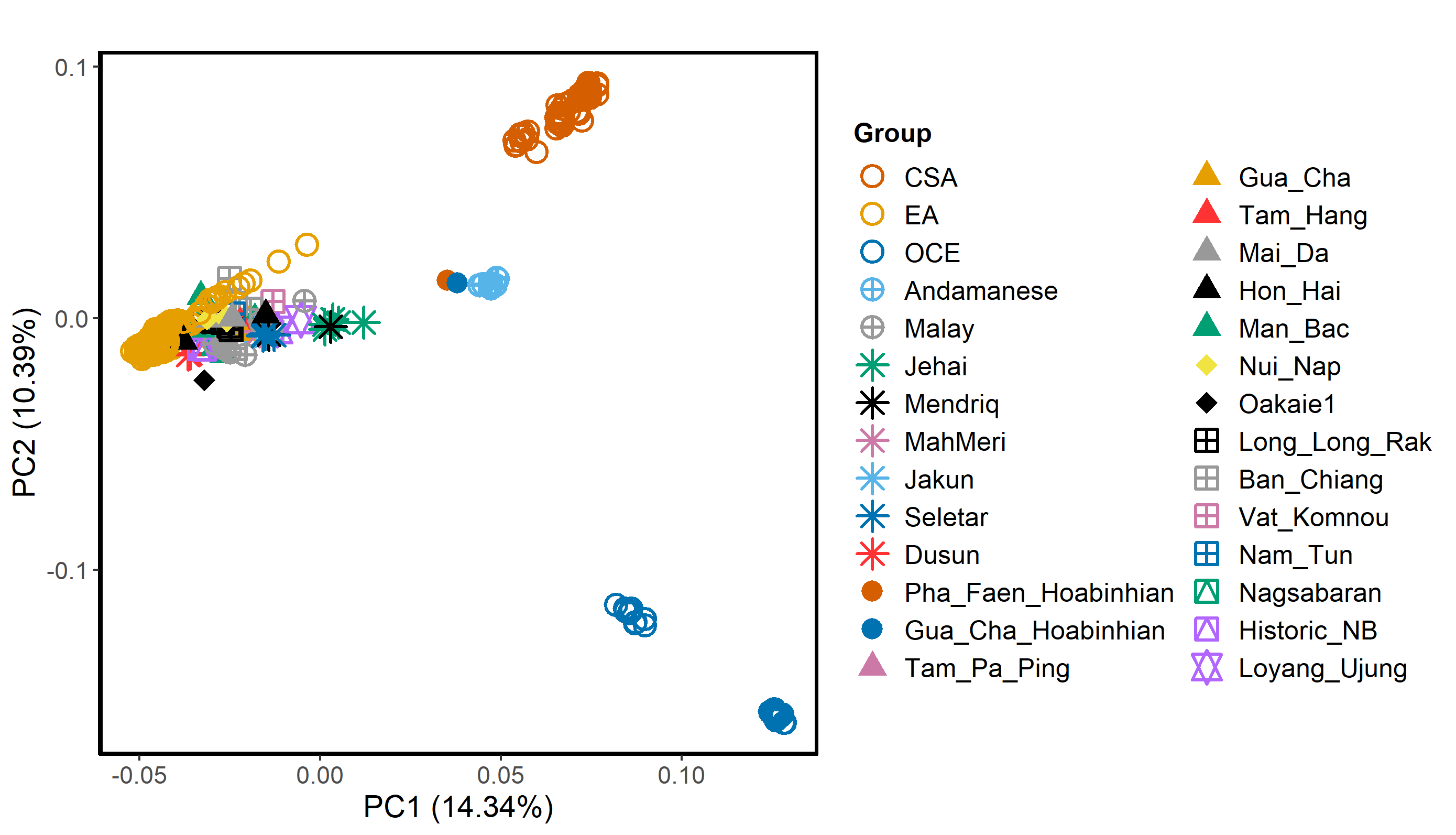


Fig S5: Cross validation error of ADMIXTURE analysis of indigenous Malaysian, Andamanese, Malay and EA, CSA, and OCE from HGDP.


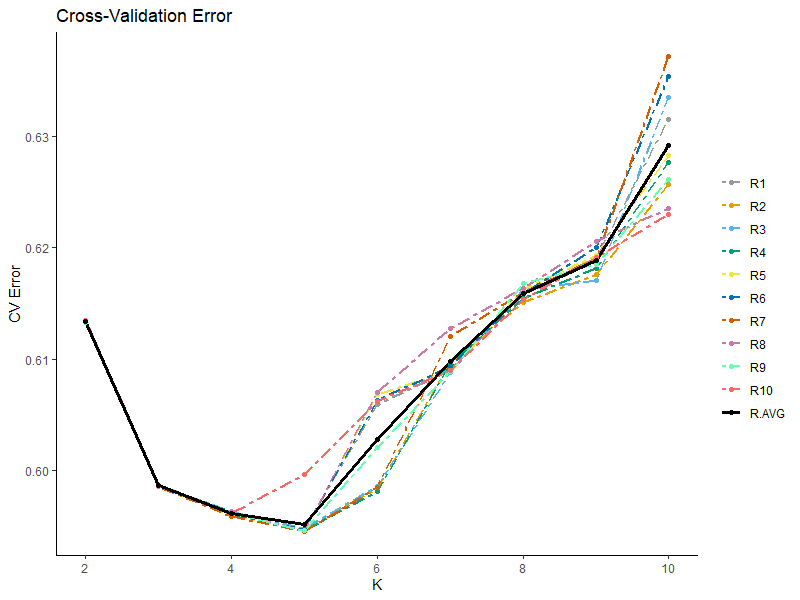


Fig S6: ADMIXTURE plot of indigenous Malaysian, Andamanese, Malay and EA, CSA, and OCE from HGDP.


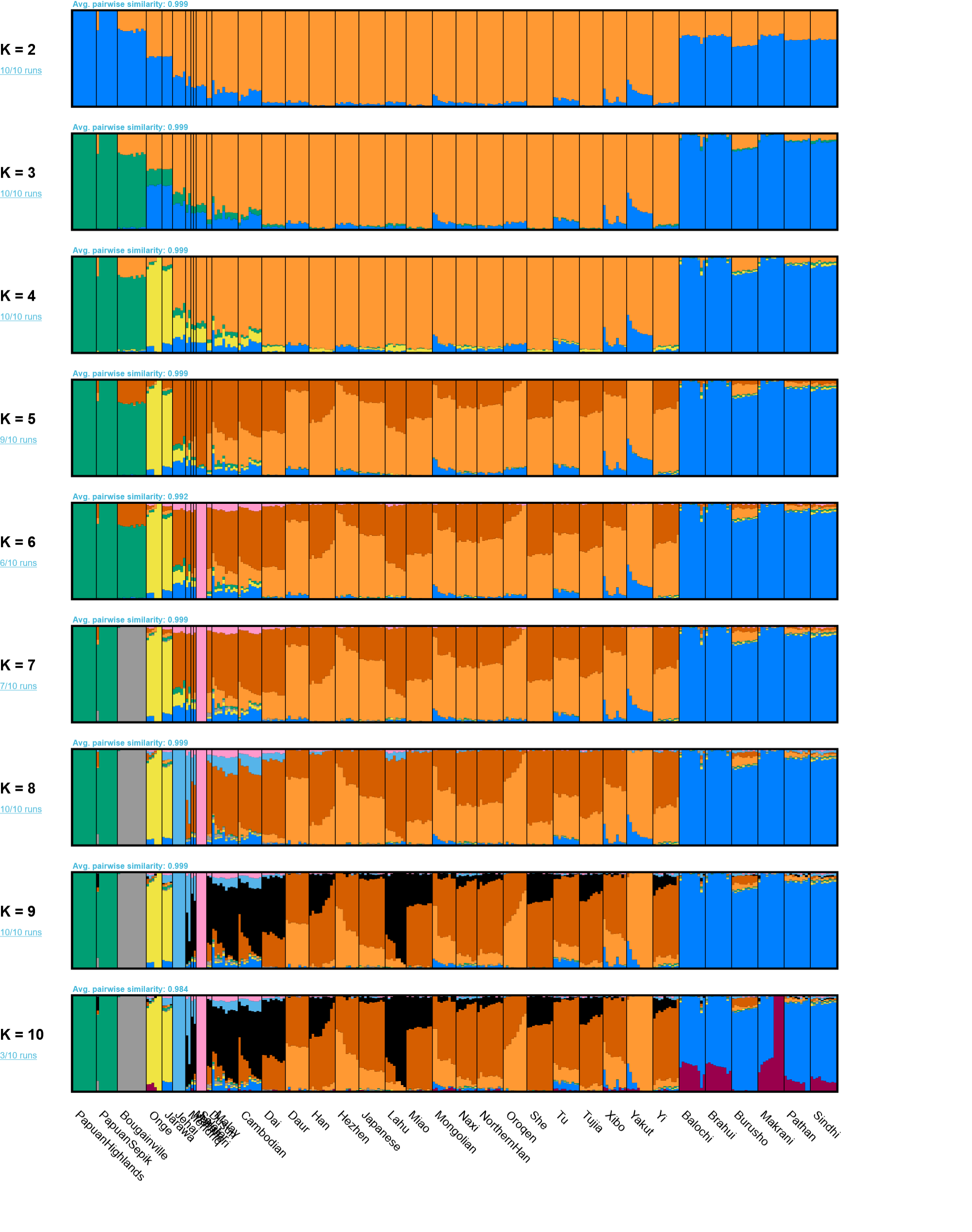


Fig S7: Cross validation error of ADMIXTURE analysis of ancient SEA, indigenous Malaysian, Andamanese, Malay and EA, CSA, and OCE from HGDP.


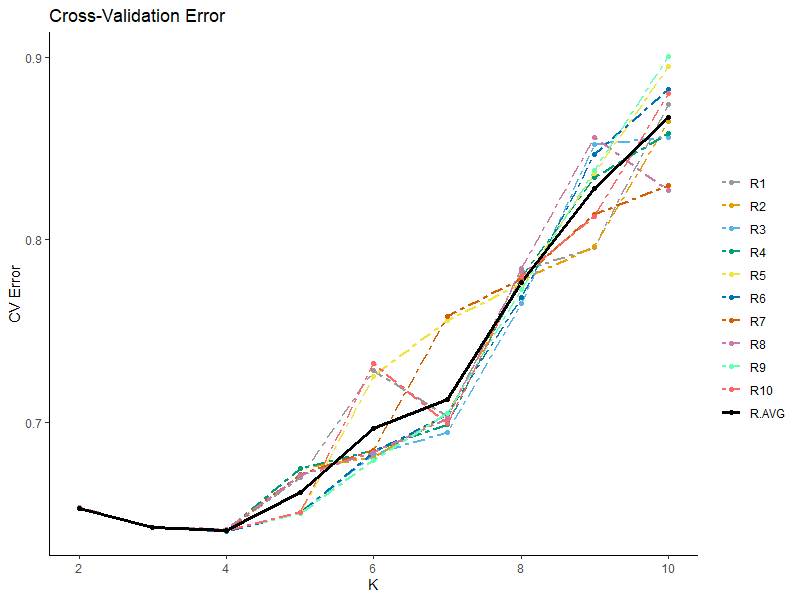


Fig S8: ADMIXTURE plot of ancient SEA, indigenous Malaysian, Andamanese, Malay and EA, CSA, and OCE from HGDP.


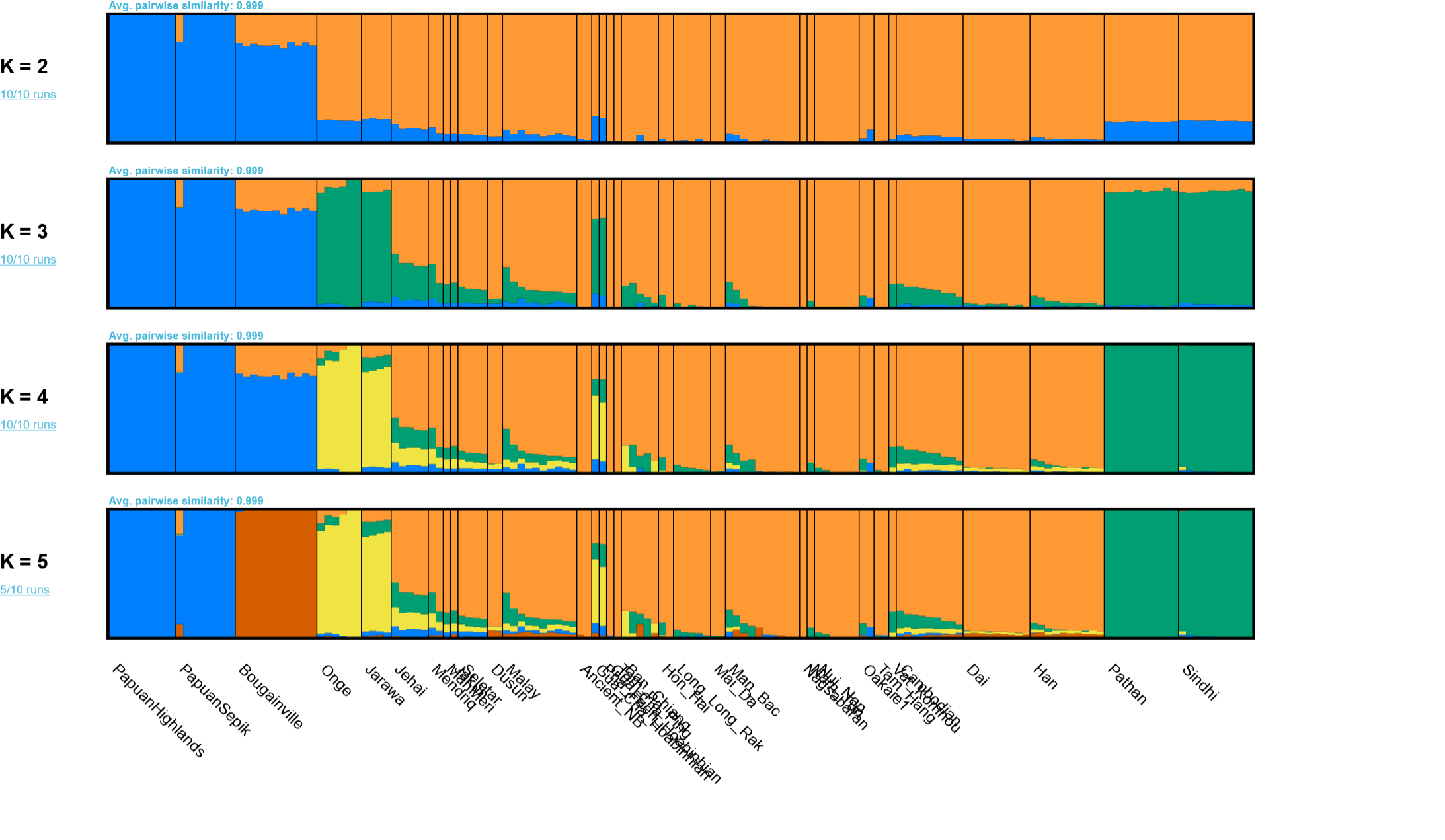


Fig S9: Inference of the effective population size (Ne) in indigenous Malaysian.


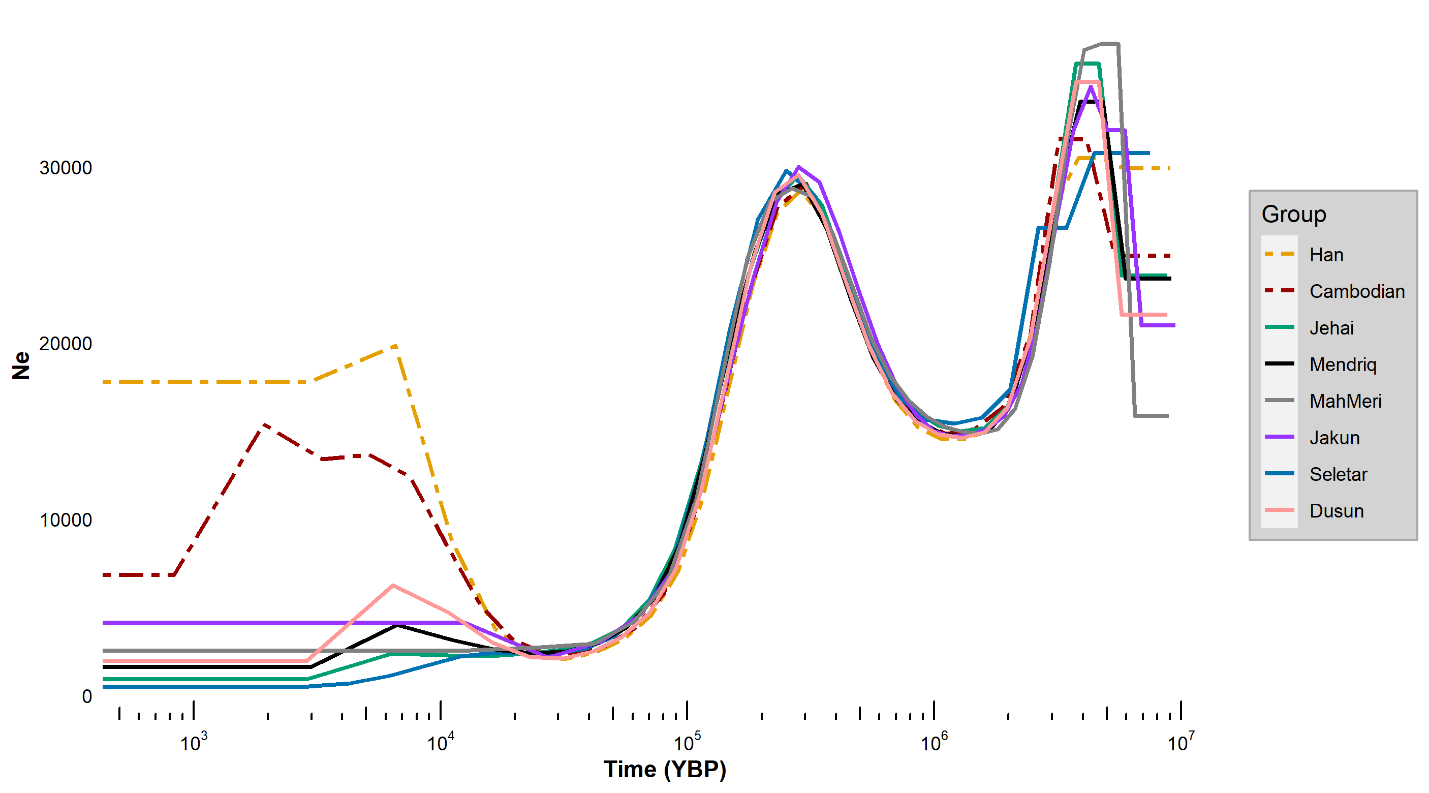


Fig S10: Estimation of divergence time between indigenous Malaysian and HGDP.


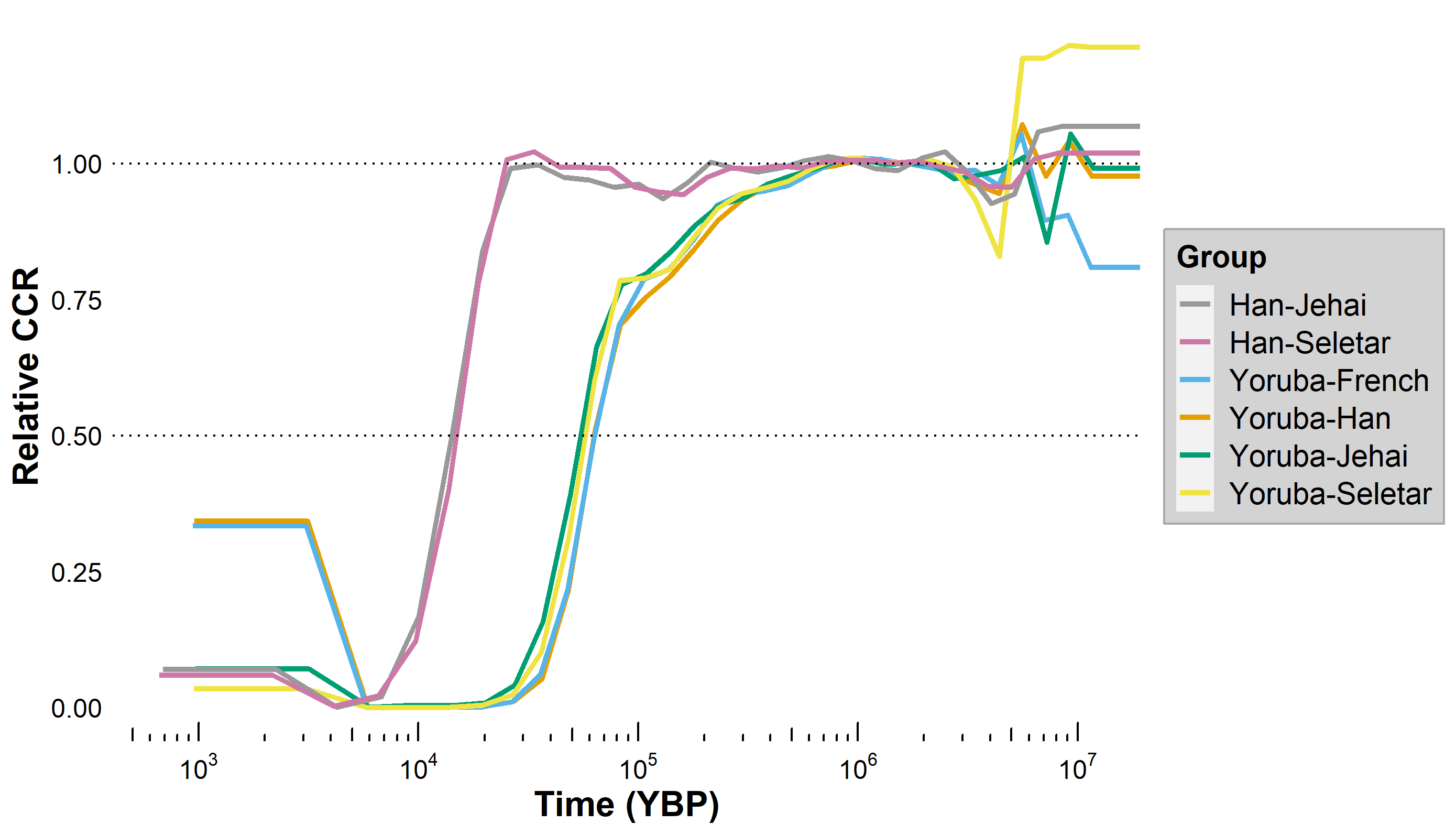


Fig S11: Cumulative Runs of Homozygosity (ROH) in selected Orang Asli and HGDP populations.


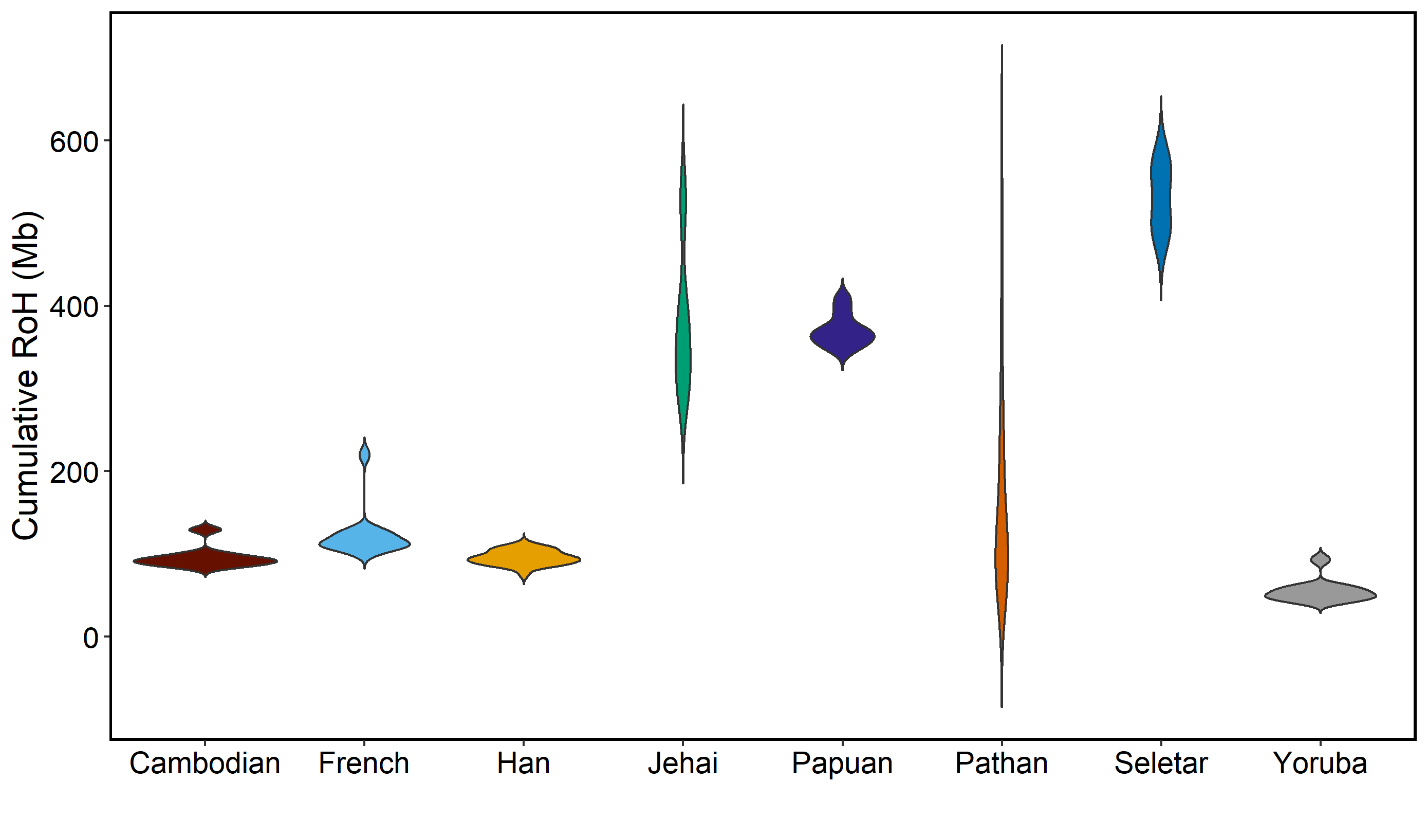


Fig S12: TreeMix maximum likelihood tree with five migration events of indigenous Malaysian and modern populations.


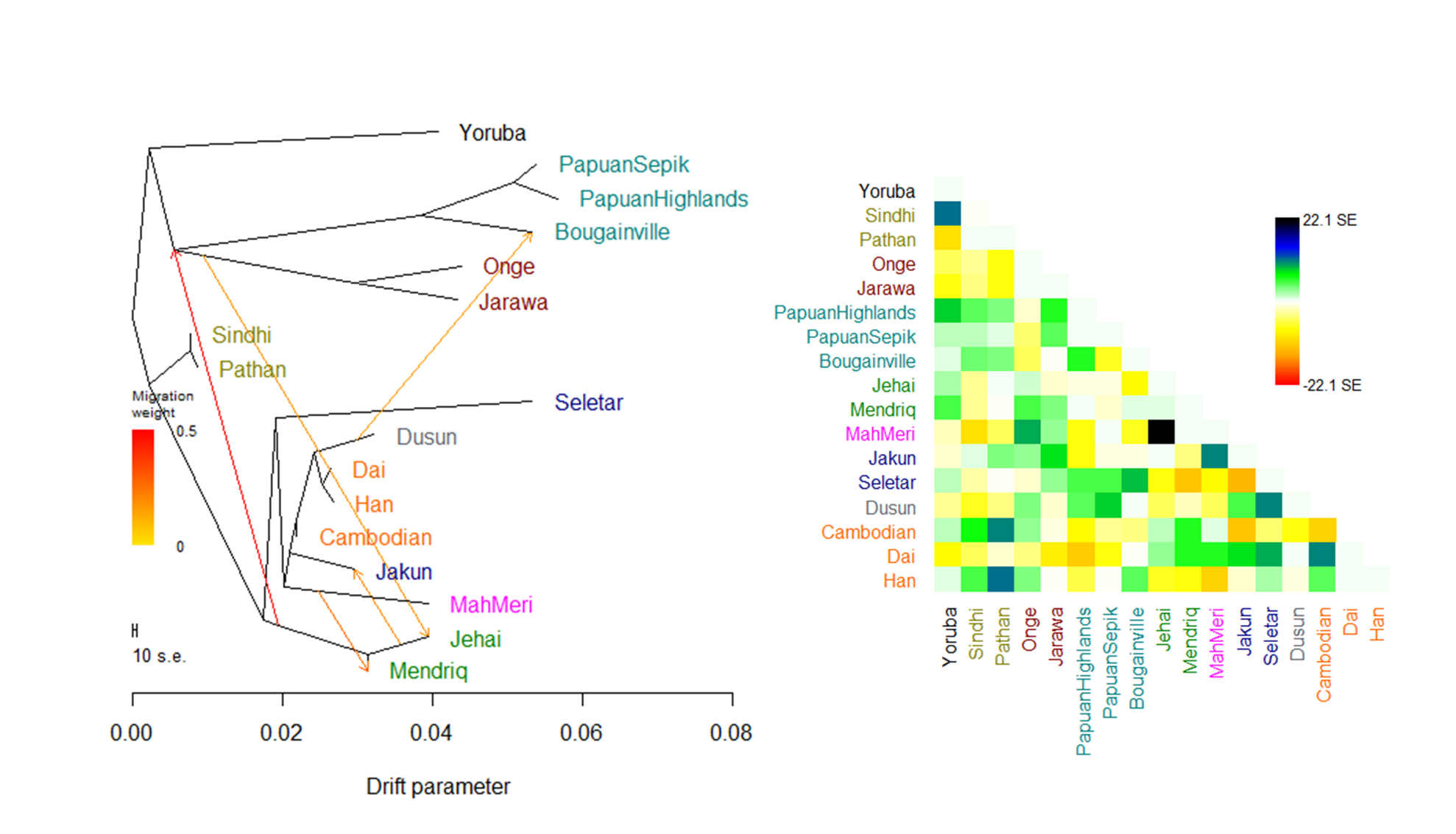


Table S1: List of samples sequenced for this study.

| Sample | Tribe | Group | Life style | Geographic location |
| --- | --- | --- | --- | --- |
| JEH02 | Jehai | Negrito | Hunter-gatherer | Perak |
| JEH03 | Jehai | Negrito | Hunter-gatherer | Perak |
| JEH04 | Jehai | Negrito | Hunter-gatherer | Perak |
| JEH05 | Jehai | Negrito | Hunter-gatherer | Perak |
| JEH06 | Jehai | Negrito | Hunter-gatherer | Perak |
| MHM02 | MahMeri | Senoi | Slash-and-Burn Farmer | Selangor |
| SLT01 | Seletar | Proto-Malay | Sea Nomad | Johor |
| SLT02 | Seletar | Proto-Malay | Sea Nomad | Johor |
| SLT03 | Seletar | Proto-Malay | Sea Nomad | Johor |
| SLT04 | Seletar | Proto-Malay | Sea Nomad | Johor |
| JKN040 | Jakun | Proto-Malay | Farmer | Pahang |

Table S2: List of Ancient Southeast Asian samples used in this study.

| Sample | Date (YBP) | Site | Country | Period | Study |
| --- | --- | --- | --- | --- | --- |
| VN22 | 3835–3695 | Man Bac | Vietnam | Neolithic | Lipson et al (2018) |
| VN29 | 3900–3600 | Man Bac | Vietnam | Neolithic | Lipson et al (2018) |
| VN31 | 3900–3600 | Man Bac | Vietnam | Neolithic | Lipson et al (2018) |
| VN33 | 3900–3600 | Man Bac | Vietnam | Neolithic | Lipson et al (2018) |
| VN34 | 4080–3845 | Man Bac | Vietnam | Neolithic | Lipson et al (2018) |
| VN37 | 3825–3635 | Man Bac | Vietnam | Neolithic | Lipson et al (2018) |
| VN39 | 3830–3695 | Man Bac | Vietnam | Neolithic | Lipson et al (2018) |
| VN40 | 3820–3615 | Man Bac | Vietnam | Neolithic | Lipson et al (2018) |
| VN41 | 2100–1900 | Nui Nap | Vietnam | Bronze Age | Lipson et al (2018) |
| VN42 | 1995–1900 | Nui Nap | Vietnam | Bronze Age | Lipson et al (2018) |
| OAI1/S28 | 3200–2700 | Oakaie 1 | Myanmar | Late Neolithic/Bronze Age | Lipson et al (2018) |
| OAI1/S29 | 3200–2700 | Oakaie 1 | Myanmar | Late Neolithic/Bronze Age | Lipson et al (2018) |
| BCES B67 | 3500–3200 | Ban Chiang | Thailand | Late Neolithic/Bronze Age | Lipson et al (2018) |
| BCES B38 | 3200–3000 | Ban Chiang | Thailand | Bronze Age | Lipson et al (2018) |
| BCES B54 | 3200–3000 | Ban Chiang | Thailand | Bronze Age | Lipson et al (2018) |
| BCES B27 | 3000–2800 | Ban Chiang | Thailand | Bronze Age | Lipson et al (2018) |
| BCES B16 | 2600–2400 | Ban Chiang | Thailand | Iron Age | Lipson et al (2018) |
| AB40 | 1890–1730 | Vat Komnou | Cambodia | Iron Age | Lipson et al (2018) |
| La368 | 7950-7794 | Pha Faen | Laos | Hunter-gatherer, Hoabinhian | McColl et al (2018) |
| Ma911 | 4415-4160 | Gua Cha Cave | Malaysia | Hunter-gatherer, Hoabinhian | McColl et al (2018) |
| Vt833 | 4291-4006 | Mai Da Dieu | Vietnam | Late Neolithic | McColl et al (2018) |
| Vt880 | ~4000 | Hon Hai Co Tien | Vietnam | Late Neolithic | McColl et al (2018) |
| La364 | 3071-2880 | Tam Pa Ping | Laos | Late Neolithic/Bronze Age | McColl et al (2018) |
| Vt778 | 2750-2500 | Nam Tun | Vietnam | Late Neolithic | McColl et al (2018) |
| Ma912 | 2690-2349 | Gua Cha Cave | Malaysia | Late Neolithic farmer | McColl et al (2018) |
| La727 | 2378-2184 | Tam Hang | Laos | Recent intrusion into Hoabinhian | McColl et al (2018) |
| La898 | ~2000 | Tam Hang | Laos | Recent intrusion into Hoabinhian | McColl et al (2018) |
| Vt777 | 2349-2180 | Mai Da Dieu | Vietnam | Late Neolithic | McColl et al (2018) |
| Vt808 | 2343-2158 | Nui Nap | Vietnam | Bronze Age | McColl et al (2018) |
| Vt781 | 2340-2158 | Nui Nap | Vietnam | Bronze Age | McColl et al (2018) |
| Vt779 | 2336-2157 | Nui Nap | Vietnam | Bronze Age | McColl et al (2018) |
| In662 | 2304-2048 | Loyang Ujung Cave | Indonesia | Late Neolithic | McColl et al (2018) |
| Vt796 | 2303-2041 | Nui Nap | Vietnam | Bronze Age | McColl et al (2018) |
| In661 | 1925-1818 | Loyang Ujung Cave | Indonesia | Late Neolithic | McColl et al (2018) |
| Phl534 | 1880-1730 | Nagsabaran | Phillipines | Red-slipped pottery - Austronesian | McColl et al (2018) |
| Th519 | 1815-1625 | Long Long Rak | Thailand | Iron Age | McColl et al (2018) |
| Th521 | 1813-1620 | Long Long Rak | Thailand | Iron Age | McColl et al (2018) |
| Th703 | 1732-1571 | Long Long Rak | Thailand | Iron Age | McColl et al (2018) |
| Th530 | 1730-1570 | Long Long Rak | Thailand | Iron Age | McColl et al (2018) |
| Th531 | 1691-1537 | Long Long Rak | Thailand | Iron Age | McColl et al (2018) |
| Ma554 | 505-326 | Supu Hujung4 | Malaysia | Historical | McColl et al (2018) |
| Ma555 | 452 299 | Kinabatagan | Malaysia | Historical | McColl et al (2018) |
| Vt719 | 307 | Hon Hai Co Tien | Vietnam | Late Neolithic | McColl et al (2018) |

Table S3: Y-chromosome and mtDNA haplogroups in indigenous Malaysian.

| Group | Tribe | Sample | MT haplogroup  (haplogrep) | MT haplogroup (MitoSuite) | Y haplogroup  (Yhaplo) |
| --- | --- | --- | --- | --- | --- |
| Negrito  Hunter-Gatherer | Jehai | JEH02 | R21 | R21 | R2a-L266 |
|  |  | JEH03 | R21 | R21 | - |
|  |  | JEH04 | M13b1 | M13b1 | R1a1a1b2a-Z94 |
|  |  | JEH05 | M21a | M21a | - |
|  |  | JEH06 | M17a | M17a | K2b-P331 |
|  | Mendriq | MNQ01 | M21a | M21a | K2b1-P397 |
|  |  | MNQ02 | F1a1a | F1a1a | - |
| North Borneo | Dusun | DSN01 | M7c1c3 | M7c1c3 | - |
|  |  | DSN02 | R9c1a | R9c1a1 | - |
| Senoi | MahMeri | MHM02 | N22a | N22a | - |
| Proto-Malay | Jakun | JKN040 | E1a2 | E1 | - |
|  | Seletar  (Sea nomads) | SLT01 | N9a6b | N9a6b | - |
|  |  | SLT02 | N9a6b | N9a6b | O2b1-F1150 |
|  |  | SLT03 | N9a6b | N9a6b | K2b1-P397 |
|  |  | SLT04 | N9a6b | N9a6b | - |

Table S4: F4 test of indigenous Malaysian and neighboring populations.

| F4(Mbuti,Onge;X,Han) | | | | | | |
| --- | --- | --- | --- | --- | --- | --- |
| Pop1 | Pop2 | Pop3 | Pop4 | F4 | SE | Z |
| Mbuti | Onge | Jehai | Han | -0.00098 | 0.000266 | -3.669 |
| Mbuti | Onge | Mendriq | Han | -0.00051 | 0.000305 | -1.662 |
| Mbuti | Onge | MahMeri | Han | -0.00024 | 0.000407 | -0.577 |
| Mbuti | Onge | Jakun | Han | -0.00014 | 0.000375 | -0.384 |
| Mbuti | Onge | Seletar | Han | 0.000462 | 0.000269 | 1.719 |
| Mbuti | Onge | Dusun | Han | -0.00014 | 0.000266 | -0.539 |
| F4(Mbuti,Jarawa;X,Han) | | | | | | |
| Pop1 | Pop2 | Pop3 | Pop4 | F4 | SE | Z |
| Mbuti | Jarawa | Jehai | Han | -0.00106 | 0.000271 | -3.921 |
| Mbuti | Jarawa | Mendriq | Han | -0.00063 | 0.000289 | -2.168 |
| Mbuti | Jarawa | MahMeri | Han | -0.0001 | 0.000409 | -0.255 |
| Mbuti | Jarawa | Jakun | Han | -0.00058 | 0.000391 | -1.478 |
| Mbuti | Jarawa | Seletar | Han | 0.000033 | 0.000295 | 0.112 |
| Mbuti | Jarawa | Dusun | Han | -0.00011 | 0.000278 | -0.411 |

| F4(Mbuti,PapuanHighlands;X,Han) | | | | | | |
| --- | --- | --- | --- | --- | --- | --- |
| Pop1 | Pop2 | Pop3 | Pop4 | F4 | SE | Z |
| Mbuti | PapuanHighlands | Jehai | Han | -0.00062 | 0.000318 | -1.952 |
| Mbuti | PapuanHighlands | Mendriq | Han | -0.000708 | 0.000333 | -2.122 |
| Mbuti | PapuanHighlands | MahMeri | Han | 0.000157 | 0.000448 | 0.35 |
| Mbuti | PapuanHighlands | Jakun | Han | -0.000113 | 0.000417 | -0.271 |
| Mbuti | PapuanHighlands | Seletar | Han | -0.000304 | 0.00033 | -0.92 |
| Mbuti | PapuanHighlands | Dusun | Han | -0.000653 | 0.000289 | -2.26 |
| F4(Mbuti,Bougainville;X,Han) | | | | | | |
| Pop1 | Pop2 | Pop3 | Pop4 | F4 | SE | Z |
| Mbuti | Bougainville | Jehai | Han | 0.000619 | 0.000283 | 2.187 |
| Mbuti | Bougainville | Mendriq | Han | 0.000872 | 0.000315 | 2.77 |
| Mbuti | Bougainville | MahMeri | Han | 0.001274 | 0.000426 | 2.987 |
| Mbuti | Bougainville | Jakun | Han | 0.000777 | 0.000381 | 2.04 |
| Mbuti | Bougainville | Seletar | Han | 0.000823 | 0.000289 | 2.845 |
| Mbuti | Bougainville | Dusun | Han | -0.00087 | 0.000269 | -3.23 |

| Pop1 | Pop2 | Pop3 | Pop4 | F4 | SE | Z |
| --- | --- | --- | --- | --- | --- | --- |
| Mbuti | Jehai | MahMeri | Han | -0.001341 | 0.000382 | -3.509 |
| Mbuti | Jehai | Jakun | Han | -0.003729 | 0.000428 | -8.723 |
| Mbuti | Jehai | Seletar | Han | 0.000226 | 0.000286 | 0.792 |
| Mbuti | Jehai | Dusun | Han | -0.000257 | 0.00026 | -0.987 |
| Mbuti | Mendriq | MahMeri | Han | -0.001492 | 0.000443 | -3.366 |
| Mbuti | Mendriq | Jakun | Han | -0.002086 | 0.000438 | -4.762 |
| Mbuti | Mendriq | Seletar | Han | 0.000734 | 0.00034 | 2.161 |
| Mbuti | Mendriq | Dusun | Han | -0.000389 | 0.000309 | -1.259 |
| Mbuti | MahMeri | Jakun | Han | -0.00054 | 0.000606 | -0.891 |
| Mbuti | MahMeri | Seletar | Han | 0.001121 | 0.000437 | 2.568 |
| Mbuti | MahMeri | Dusun | Han | -0.000474 | 0.000374 | -1.268 |

| Pop1 | Pop2 | Pop3 | Pop4 | F4 | SE | Z |
| --- | --- | --- | --- | --- | --- | --- |
| Mbuti | Ma911 | Jehai | Han | -0.003033 | 0.000506 | -5.992 |
| Mbuti | Ma911 | Mendriq | Han | -0.002242 | 0.000561 | -3.999 |
| Mbuti | Ma911 | MahMeri | Han | -0.001206 | 0.000724 | -1.667 |
| Mbuti | Ma911 | Jakun | Han | 0.000242 | 0.000703 | 0.344 |
| Mbuti | Ma911 | Seletar | Han | 0.000022 | 0.000552 | 0.04 |
| Mbuti | Ma911 | Dusun | Han | 0.000537 | 0.000525 | 1.023 |
| Mbuti | Jehai | Ma912 | Han | -0.001721 | 0.000378 | -4.549 |
| Mbuti | Mendriq | Ma912 | Han | -0.00223 | 0.000441 | -5.057 |
| Mbuti | MahMeri | Ma912 | Han | -0.002213 | 0.000592 | -3.738 |
| Mbuti | Jakun | Ma912 | Han | 0.000175 | 0.000556 | 0.315 |
| Mbuti | Seletar | Ma912 | Han | 0.000336 | 0.000441 | 0.76 |
| Mbuti | Dusun | Ma912 | Han | 0.003013 | 0.000459 | 6.569 |
